# Comparison of three *Agrobacterium*-mediated co-transformation methods for generating marker-free transgenic *Brassica napus* plants

**DOI:** 10.1186/s13007-020-00628-y

**Published:** 2020-06-05

**Authors:** Fang Liu, Pandi Wang, Xiaojuan Xiong, Ping Fu, Hongfei Gao, Xinhua Ding, Gang Wu

**Affiliations:** 1 Key Laboratory of Biology and Genetics Improvement of Oil Crops, Ministry of Agriculture and Rural Affairs, Oil Crops Research Institute, Chinese Academy of Agricultural Sciences, Wuhan, China; 2 State Key Laboratory of Crop Biology, College of Plant Protection, Shandong Agricultural University, Tai’an, 271018 Shandong China

**Keywords:** *Brassica napus*, Co-transformation, “Double T-DNA” vector system, Herbicide resistant, Marker-free, Mixed-strain system

## Abstract

**Background:**

Generation of marker-free transgenic plants is very important to the regulatory permission and commercial release of transgenic crops. Co-transformation methods that enable the removal of selectable marker genes have been extensively used because they are simple and clean. Few comparisons are currently available between different strain/plasmid co-transformation systems, and also data are related to variation in co-transformation frequencies caused by other details of the vector design.

**Results:**

In this study, we constructed three vector systems for the co-transformation of allotetraploid *Brassica napus* (*B. napus*) mediated by *Agrobacterium tumefaciens* and compared these co-transformation methods. We tested a mixed-strain system, in which a single T-DNA is harbored in two plasmids, as well as two “double T-DNA” vector systems, in which two independent T-DNAs are harbored in one plasmid in a tandem orientation or in an inverted orientation. As confirmed by the use of PCR analysis, test strips, and Southern blot, the average co-transformation frequencies from these systems ranged from 24 to 81% in T_0_ plants, with the highest frequency of 81% for 1:1 treatment of the mixed-strain system. These vector systems are valuable for generating marker-free transgenic *B. napus* plants, and marker-free plants were successfully obtained in the T_1_ generation from 50 to 77% of T_0_ transgenic lines using these systems, with the highest frequency of 77% for “double T-DNA” vector systems of pBID RT Enhanced. We further found that marker-free *B. napus* plants were more frequently encountered in the progeny of transgenic lines which has only one or two marker gene copies in the T_0_ generation. Two types of herbicide resistant transgenic *B. napus* plants, *Bar*^+^ with phosphinothricin resistance and *Bar*^+^*EPSPS*^+^*GOX*^+^ with phosphinothricin and glyphosate resistance, were obtained.

**Conclusion:**

We were successful in removing selectable marker genes in transgenic *B. napus* plants using all three co-transformation systems developed in this study. It was proved that if a appropriate mole ratio was designed for the specific length ratio of the twin T-DNAs for the mixed-strain method, high unlinked co-insertion frequency and overall success frequency could be achieved. Our study provides useful information for the construction of efficient co-transformation system for marker-free transgenic crop production and developed transgenic *B. napus* with various types of herbicide resistance.

## Background

Allotetraploid oilseed rape *Brassica napus* L. (*B. napus*) is one of the most important sources of plant oil and it is a major crop used for generating protein-rich products worldwide. To meet the increasing demand, work has been undertaken to improve existing cultivars and to generate new elite cultivars of *B. napus* using genetic engineering approaches, which provide an alternative approach to creating novel varieties with improved traits and desirable characteristics that can’t be obtained by traditional breeding methods [1]. Genetic transformation techniques have been used successfully for *B. napus* improvement by employing an *Agrobacterium tumefaciens* (*A. tumefaciens*)-mediated system [2, 3] since the first reports of successful *B. napus* transformation [4, 5]. Many important traits, including herbicide [6], insect [7] and fungal [8] resistance, as well as improved composition of oil [9, 10] and proteins [11] have been introduced into *B. napus* by transgenic methods. The safety of transgenic *B. napus* is facing the same questions and debates as for other transgenic crops, and one concern is over the continued presence of a selectable marker gene in the final plant product [12, 13].

Gene transfer requires the simultaneous transformation of a antibiotic or herbicide resistance selectable marker gene to select positive transgenic plants carrying the gene of interest. Once the transgenic plants have been regenerated and characterized, the selectable marker gene is no longer necessary. Moreover, transgenic plants with marker genes not only impair public acceptance due to the concerns of public health but also increase the environmental risk of the introgression of marker genes into weedy relatives and non-transgenic crops [14]. These factors complicate the regulatory process for the commercialization of genetically modified plants [15, 16]. In addition, marker genes limit the process of gene stacking through re-transformation [16]. Therefore, producing marker-free transgenic *B. napus* varieties, where the marker gene has been eliminated, will be beneficial to their development and eventual commercial release. Genetically modified *B. napus* varieties without marker genes have been commercially released in a small number of countries, including the United States, Canada, Australia and Chile thus far [17].

To date, a number of methods that enable the removal of selectable marker genes have been developed, including co-transformation [18, 19], site-specific recombination [20–27], homologous recombination [28], transposon-mediated repositioning [29] and gene editing [30]. Among these methods, co-transformation has been extensively used because it is a simple and clean technique and it doesn’t leave behind residual DNA sequences such as invert repeats and recombination sites in transgenic plants from which the selectable marker gene has been eliminated with a high frequency [16, 31, 32]. Co-transformation involves the simultaneous integration of a selectable marker gene and a gene of interest from different T-DNAs, as well as their subsequent recombination and segregation in the progeny, if the two genes are integrated into unlinked loci [31, 33]. Co-transformation has been applied in many species, including soya bean [34], tobacco [35], maize [18], sorghum [36], rice [37], durum wheat [19] and *B. napus* [38]. Elimination of selectable marker genes after co-transformation can be realized by using a single *Agrobacterium* strain system with a plasmid that contains two independent T-DNA regions (the one strain/one plasmid method or the “double T-DNA” vector system) [35], or by using a two *Agrobacterium* strain system harboring two plasmids that each contain an independent T-DNA (the two strains/two plasmids method or the mixed-strain system) [38]. Zhou et al. [39] and Komari et al. [40] reported that the “double T-DNA” vector system might be more effective than the mixed-strain method in producing co-transformants.

Although co-transformation strategies have been developed over the past 30 years, it is more popular in model plants, such as tobacco and rice, than in other plants. Co-transformation strategies have not yet been applied widely in *B. napus,* and there are few comparisons between different strain/plasmid systems, and also few studies examine variation in co-transformation frequencies caused by other details of the vector design studies. In order to obtain guidelines as to how to construct an efficient co-transformation system to produce marker-free *B. napus*, we established three systems and provide a comprehensive and scientific comparison of these co-transformation systems in the same *Agrobacterium*-mediated transformation platform with respect to *B. napus* and evaluated these systems with the same criteria. In the first system, the two strains/two plasmids method was applied, and the different strains were mixed in a series of ratios. In the second and third systems, the one strain/one plasmid method was employed. Double T-DNAs, arrayed in a tandem orientation or in an inverted orientation relative to each other, were introduced into one binary vector and were carried by one *Agrobacterium* strain (Table 1). Gene of interest and selectable marker gene from different T-DNAs were able to be simultaneously integrated in T_0_ and marker-free plants can be yielded after their subsequent recombination and segregation in T_1_. We examined the combination of co-transformation and subsequent marker-free efficacy by studying the molecular and genomic organization of the transgenic plants. Our results indicate that the systems developed in this study were effective in removing selectable marker genes, in this particular case herbicide resistant *B. napus* plants. Our study has obtained information about these co-transformation systems which will be valuable in future *B. napus* breeding projects using genetic engineering approaches.

**Table 1:** Information of the three co-transformation vector systems

Classification of system	Vector	Gene of interest	Selectable marker gene
Mixed-strain system	pCAMBIA1300 and pCAMBIA3300	*Bar*	*HPT*
“Double T-DNA” system	pDB1300-3300	*Bar*	*HPT*
	pBID RT Enhanced	*Bar*	*EPSPS*

## Methods

### Plant material

The allotetraploid *B. napus* genotype used for genetic transformation in this study is Zhongshuang 6 (ZS6), a semi-winter variety provided by the Oil Crops Research Institute of Chinese Academy of Agricultural Sciences in Wuhan, China. ZS6 is an elite Chinese cultivar due to its low erucic acid and glucosinolate levels and high oil content (39.08%). Transgenic plants and the wild type were grown in pots containing a mixture of moss peat (PINDSTRUP, Denmark) and field soil (3:1). Plants were maintained in a greenhouse under growth conditions of 20 °C ± 2 °C under a 16/8 h photo-period at a light intensity of 44 umol m^−2^ s^−1^ and 60–90% relative humidity.

### Expression vectors with two independent T-DNA regions

**Fig. 1:**
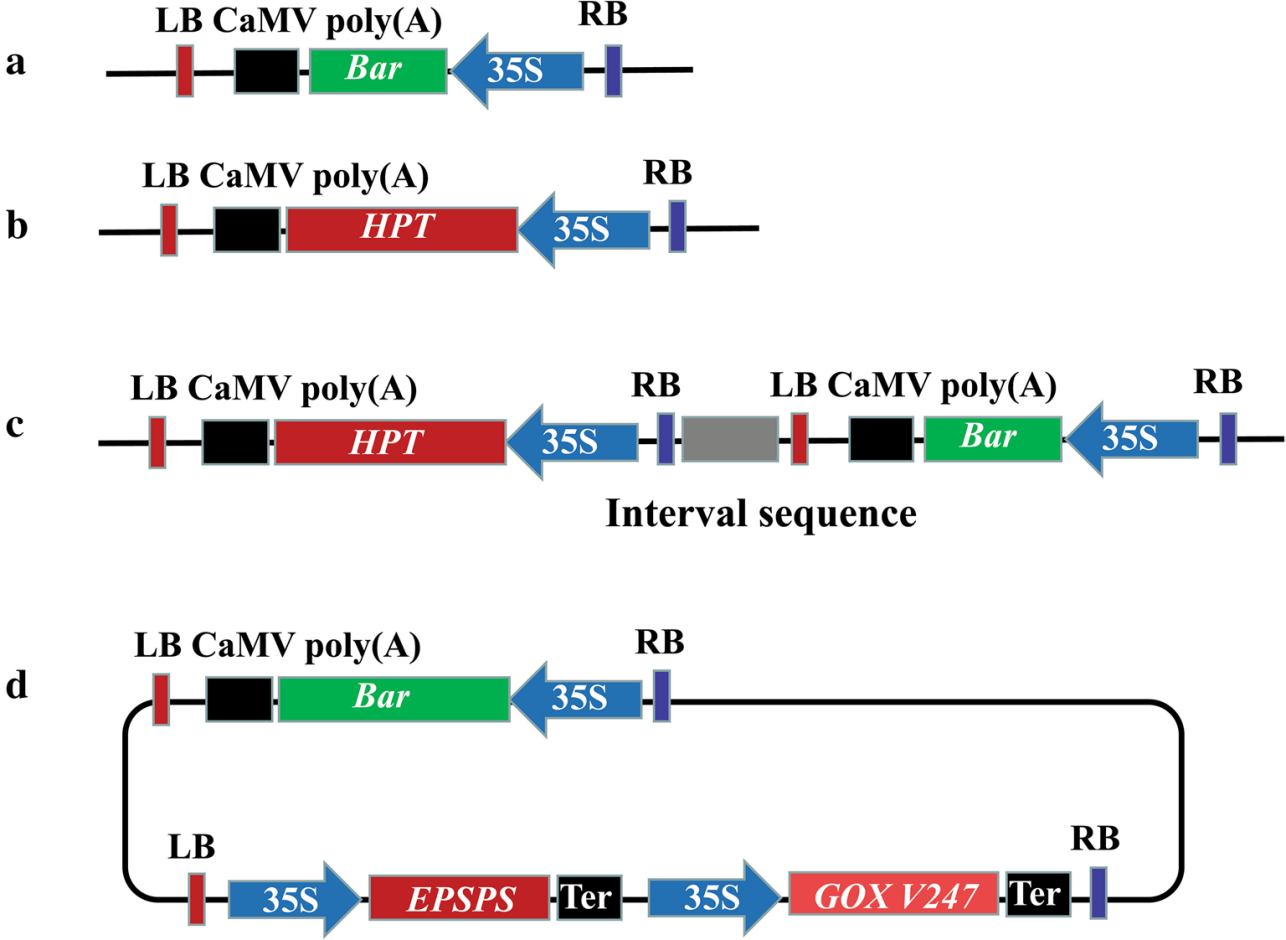
Vector maps of three co-transformation systems. The mixed-strain system includes vectors of pCAMBIA3300 (**a**) and pCAMBIA1300 (**b**) showing the independent T-DNA region with *Bar* and *HPT* expression cassettes. The “double T-DNA” vector system of pDB1300-3300 (**c**) shows two T-DNA regions with the *Bar* and *HPT* expression cassettes. The “double T-DNA” vector system of pBID RT Enhanced (**d**) shows two T-DNA regions with *Bar*, *EPSPS* and *GOX V247* expression cassettes

For the mixed-strain system, two commercial expression vectors, pCAMBIA1300 and pCAMBIA3300 (https://cambia.org/, YOUBIO, Hunan, China), were used for delivering two T-DNAs from mixtures of *A. tumefaciens* strains, each containing only one T-DNA. They are commonly used binary expression vectors in plant genetic engineering and are harmless in *E. coli*, *Agrobacterium* and plants [41]. pCAMBIA1300 contained one T-DNA with the *Hygromycin phosphotransferase* (*HPT*) gene, which was under the control of the CaMV35S promoter and followed by the CaMV poly(A) signal sequence. pCAMBIA3300 harbored one T-DNA region with the phosphinothricin N-acetyltransferase gene (*Bar*), which is under the control of the same CaMV35S promoter and followed by the CaMV poly(A) signal (Fig. 1a, b, Additional file 2: Fig. S1). In this study, the *Bar* gene of pCAMBIA3300 was regarded as a gene of interest and the *HPT* gene of pCAMBIA1300 as a selectable marker gene (Table 1).

The “Double T-DNA” binary vector pDB1300-3300, which carried two independent T-DNAs in a tandem orientation, with one T-DNA containing a selectable marker *HPT* gene and the other a *Bar* gene (Table 1, Fig. 1c), was constructed as follows: the plasmid pCAMBIA1300 was double digested with EcoRI (Thermo Fisher Scientific, America) and SacI (Thermo Fisher Scientific, America). A 387 bp fragment of the *GUS* (*β*-*glucuronidase*) gene, which was used as an interval sequence between the left border and right border, was amplified from pBI121 with primers RB + GUS + LB-L: TTTGAATTCGCGGGTAAACCTAAGAGAAAAGAGCGTTTATTAGTGGGCAGATGAACATGGCAT (containing the right border sequence) and RB + GUS + LB-R: ACCGAGCTCAATTTGTTTACACCACAATATATCCTGCCACCAGATAACGGTTCAGGCACAGCA (containing the left border sequence) and then ligated to the digested pCAMBIA1300 to form an intermediate vector pCAMBIA1300-1. pCAMBIA1300-1 was then digested with BamHI and HindIII. The CaMV poly(A) signal sequence, *Bar* gene and CaMV35S promoter were amplified from pCAMBIA3300 with primers polyA + *Bar *+ 35S-L: TTTGGATCCTGACGCTTAGACAACTTAATAACACA and polyA + *Bar *+ 35S-R: TTTAAGCTTTCGTGCCAGCTGCATTAATGAA, and inserted into the digested pCAMBIA1300-1. The expression vector pDB1300-3300 harboring two T-DNA regions in a tandem orientation was thus constructed (Additional file 2: Fig. S2). All the primers were synthesized by Sangon Biotech (Shanghai, China).

The “Double T-DNA” expression vector pBID RT Enhanced contains two independent T-DNA regions in an inverted orientation, with the *Bar* gene in the first T-DNA region and the *CP4 EPSPS* and *GOX V247* genes in the second. *CP4 EPSPS* was taken as the selectable marker gene during the period of genetic transformation in this system (Fig. 1d, Table 1). *CP4 EPSPS* originates from *Salmonella typhimurium* and encodes a 5-enolpyruvate phenyloxalate-3-phosphate lipase, rendering plant resistance to herbicide glyphosate. However, glyphosate residues have some negative impacts on, e.g., sensitive pollination and reproductive development of plants [42]. This is the rationale for generating glyphosate resistant *B. napus* with the glyphosate-degrading gene *GOX V247* transferred together with the *CP4 EPSPS* gene to confer resistance to glyphosate while reducing glyphosate residues [43]. In addition to being marker-free, the plants were also expected to create single and multiple herbicide resistant *B. napus* by these systems. The vector pBID RT Enhanced was constructed on the basis of an initial skeleton of pCAMBIA3300 and an additional artificially synthesized fragment. First, plasmid pCAMBIA3300 containing the *Bar* gene was phosphorylated to ensure its multiple cloning site (MCS) was invalidated, and a new MCS was inserted between the pBR322 bom and pVS1 oriV sites. The synthetic sequence containing the left border, Enhanced CaMV 35S promoter, *CP4 EPSPS* gene, NOS terminator, Enhanced CaMV 35S promoter, *GOX V247* gene, NOS terminator and right border (Additional file 1: Table S1), with a total length of 5776 bp, was inserted into the new MCS in pCAMBIA3300. Finally, the desired expression vector, pBID RT Enhanced, was constructed (Additional file 2: Fig. S3).

### Genetic transformation

The four expression vectors were introduced into *A. tumefaciens* GV3101 (Weidi Biotechnology, Shanghai, China) by electroporation system of MicroPulser™ (Bio-Rad, America) respectively. Then etiolated hypocotyl segments of *B. napus* cv. ZS6 were transformed with *A. tumefaciens* strain GV3101 harboring the vectors. The detailed steps of this genetic transformation were described by Liu et al. [44]. Embryonic calli and regenerated shoots were screened on selection media containing 25 mg/L hygromycin (Sigma, America) for the mixed-strain method and the “double-T-DNA” vector of pDB1300-3300. For transformation of the “double-T-DNA” vector of pBID RT Enhanced, because hypocotyl segments are sensitive to glyphosate, embryonic calli grew on selection media not containing glyphosate and regenerated shoots were screened on regeneration media containing 80 mg/L glyphosate (Bayer, Germany) in order to make transformed cells more competitive than untransformed cells. Rooted shoots were transferred to soil for further analysis.

For the transformation of mixed strains containing the independent plasmids of pCAMBIA1300 or pCAMBIA3300, seven treatments were set up with varying concentration proportion of the strain harboring pCAMBIA1300 to the strain harboring pCAMBIA3300. The concentration proportions were 1:8, 1:4, 1:2, 1:1, 2:1, 4:1 and 8:1. First, each vector was introduced into *A. tumefaciens* GV3101 by electroporation system of MicroPulser™ (Bio-Rad, America), and positive clones were selected on LB agar plates at 37 °C, supplemented with appropriate concentrations of antibiotics (gentamicin: 50 mg/L, rifampicin: 50 mg/L and kanamycin: 50 mg/L, Sigma, America), and verified by PCR. A single positive *A. tumefaciens* colony containing pCAMBIA1300 or pCAMBIA3300 was obtained, and each colony was cultured in 50 mL LA medium with 50 mg/L kanamycin until the Optical Density (OD) reached 0.2. The strains were then mixed according to the concentration proportion described above with a final volume of 50 mL for infection.

### PCR-based genotyping, test strip detection of transgenic *B. napus* plants

Genomic DNA was extracted from leaves of T_0_ and T_1_ transgenic plants using a Plant Genomic DNA Kit (DP305, TIANGEN, China) following the manufacturer’s instructions. The presence of the *Bar* gene in transgenic plants was demonstrated by PCR amplification of a 440 bp fragment using the primer pair 5′-GAAGTCCAGCTGCCAGAAAC-3′ and 5′-GCACCATCGTCAACCACTAC-3′. The presence of the *HPT* gene in transgenic plants was verified by PCR amplification of a 486 bp fragment with the primer pair 5′-ACTTCTACACAGCCATCGGT-3′ and 5′-GCAAACTGTGATGGACGACA-3′. The *EPSPS* and *GOX V247* genes were evidenced by PCR amplification of a 459 bp fragment using the primer pair 5′-CGTTGAGACTGATGCTGACG-3′ and 5′-TTGAGCTTGAGACCGTTTGC-3′ as well as a 503 bp fragment using the primers 5′-TTGAGAGCACGACCTTCAGT-3′ and 5′-CCAAAGTGGCTTCTTGACCC-3′. All the primers were synthesized by Sangon Biotech (Shanghai, China) and the PCR machines were C1000™ (Bio-Rad, America).

Test strips can be used to detect protein effectively [45, 46] Putative transgenic *B. napus* plants and their self-pollinated or ZS6-crossed offspring were identified based on the detection of the Bar or CP4 EPSPS protein with test strip kits developed by the Oil Crops Research Institute, Chinese Academy of Agricultural Sciences (Wuhan, China); the procedure was carried out according to the manufacturer’s instructions.

For the assessment of T_0_ seedlings, the statistics summarized in Tables 2 and 3 were derived from the intersection of the PCR and test strip detection results. For all T_1_ seedlings from the verified T_0_ plants, the statistics in Tables 4 and 5 were based on only PCR results.

**Table 2:** Co-transformation frequencies in T_0_ transformants of the mixed-strain system and the “double T-DNA” vector system of pDB1300-3300

Strains transformed	Number of transformants				
	Rooted plants	*HPT*^+^ plants	*Bar*^+^*HPT*^+^	*Bar*^−^*HPT*^+^	Co-transformation frequency (%)
pCAMBIA1300/3300 (1:8)	58	58	24	34	41
pCAMBIA1300/3300 (1:4)	28	28	17	11	61
pCAMBIA1300/3300 (1:2)	34	34	22	12	65
pCAMBIA1300/3300 (1:1)	57	53	43	10	81
pCAMBIA1300/3300 (2:1)	90	89	38	51	43
pCAMBIA1300/3300 (4:1)	21	21	5	16	24
pCAMBIA1300/3300 (8:1)	91	87	24	63	28
Total	379	370	173	197	47
pDB1300-3300	26	26	17	9	65

**Table 3:** Co-transformation frequencies in T_0_ transformants of the “double T-DNA” system of pBID RT Enhanced determined by PCR

Strains transformed	Number of transformants				
	Rooted plants	*EPSPS*^+^ plants	*Bar*^+^*EPSPS*^+^*GOX*^+^	*Bar*^−^*EPSPS*^+^*GOX*^+^	Co-transformation frequency (%)
pBID RT Enhanced	76	57	18	39	32

**Table 4:** Genotyping detection in T_1_ transformants of the mixed-strain system and the “double T-DNA” vector system of pDB1300-3300 as detected by PCR and test strips

T_0_ events	Number of T_1_ plants					
*HPT*^+^*Bar*^+^	Total number	*Bar*^+^*HPT*^+^	*Bar*^−^*HPT*^+^	*Bar*^+^*HPT*^−^	*Bar*^−^*HPT*^−^	Marker-free plant frequency (%)
pCAMBIA1300/3300 (1:8)-10	31	26	0	1	4	3
pCAMBIA1300/3300 (1:8)-15	38	28	3	3	4	8
pCAMBIA1300/3300 (1:8)-24	13	7	6	0	0	/
pCAMBIA1300/3300 (1:4)-10	47	39	0	1	7	3
pCAMBIA1300/3300 (1:2)-1	43	37	0	0	6	/
pCAMBIA1300/3300 (1:2)-2	17	13	0	0	4	/
pCAMBIA1300/3300 (1:2)-5	35	15	9	5	6	14
pCAMBIA1300/3300 (1:1)-30	40	36	1	1	2	3
pCAMBIA1300/3300 (2:1)-30	21	18	0	0	3	/
pCAMBIA1300/3300 (8:1)-15	22	9	3	8	2	36
Average for the marker-free lines						11
pDB1300-3300-3	25	13	5	5	2	20
pDB1300-3300-11	10	10	0	0	0	/

**Table 5:** Genotype detection of T_1_ marker-free plants generated with the “double T-DNA” vector system of pBID RT Enhanced

T_0_ events	Number of T_1_ plants					
*EPSPS*^+^*Bar*^+^	Total number	*Bar*^+^*EPSPS*^+^*GOX*^+^	*Bar*^−^*EPSPS*^+^*GOX*^+^	*Bar*^+^*EPSPS*^−^*GOX*^−^	*Bar*^−^*EPSPS*^−^*GOX*^−^	Marker-free plant frequency (%)
pBID RT Enhanced-5	18	14	0	4	0	22
pBID RT Enhanced-16	12	0	1	0	11	/
pBID RT Enhanced-26	15	0	0	0	15	/
pBID RT Enhanced-27	18	11	2	1	4	6
pBID RT Enhanced-29	19	12	3	0	4	/
pBID RT Enhanced-31	17	15	1	1	0	6
pBID RT Enhanced-41	18	14	0	4	0	22
pBID RT Enhanced-65	17	10	0	7	0	41
Average for the marker-free selfed lines						19
pBID RT Enhanced-27 *ZS6	17	6	1	7	3	41
pBID RT Enhanced-29 *ZS6	23	12	2	1	8	4
pBID RT Enhanced-31 *ZS6	19	17	0	2	0	11
pBID RT Enhanced-41 *ZS6	17	8	0	9	0	53
pBID RT Enhanced-65 *ZS6	22	10	9	1	2	5
Average for the marker-free backcrossed lines						23
Average for all the marker-free lines						21

T_1_ plants were generated by selfing and by backcrossing to WT ZS6

### Southern blot analysis of transgenic *B. napus* plants

For Southern blot analysis of transgenic *B. napus* T_0_ and T_1_ plants, more than 100 μg of total genomic DNA was obtained using a standard CTAB method [47]; a total of 30 μg of DNA from each sample was digested with suitable restriction endonuclease. The digested DNA samples and DNA molecular weight marker II, with Digoxin-labelled (DIG-labelled) (Roche, Germany). were electrophoresed on 0.8% agarose gel (Biowest, Spain), transferred onto a nylon Hybond-N^+^ membrane (Roche, Germany) and hybridized with DIG-labelled probes. The hybridization and detection steps were carried out according to the instructions for the DIG High Prime DNA Labeling and Detection Starter Kit II (Roche, Germany).

DNA samples from the mixed-strain system and the “double T-DNA” system of pDB1300-3300 were all digested with *Hin*dIII (Takara, Japan), and hybridized with probes of *Bar* (262 bp) and *HPT* (556 bp). For DNA samples from the “double T-DNA” system of pBID RT Enhanced, samples digested with *Hin*dIII were hybridized with a probe of *Bar* (262 bp) and samples digested with EcoRI (Takara, Japan) were hybridized with a probe of *EPSPS* (579 bp).

## Results

### Co-transformation using the three systems resulted in different co-transformation frequencies in the T_0_ generation

**Fig. 2:**
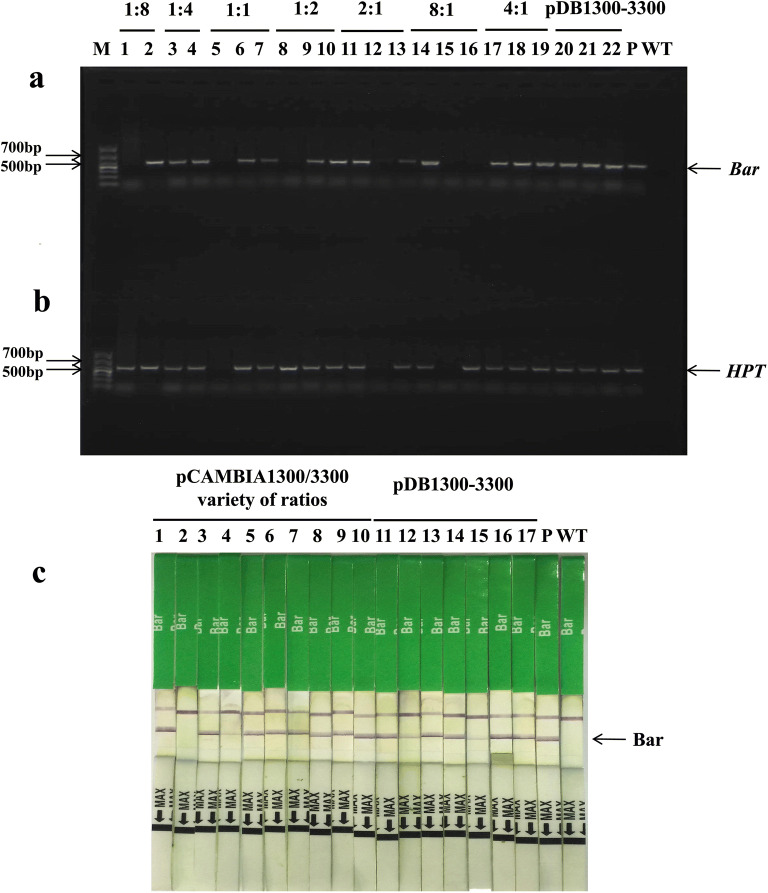
Detection of T_0_ transformants from the mixed-strain system and “double T-DNA” system of pDB1300-3300. PCR detection was done for *Bar* (**a**) and *HPT* (**b**) genes, M: DL1000 DNA marker; 1–22: individual T_0_ plants from pCAMBIA1300/3300 of a variety of ratios (X:X) and plants from pDB1300-3300); P: expression vector pCAMBIA3300 for (**a**) and pCAMBIA1300 for (**b**); WT: wild type ZS6. Test strips detection was done for the Bar protein (**c**), 1–17: individual T_0_ plants from pCAMBIA1300/3300 of a variety of ratios and plants from pDB1300-3300; P: positive transgenic *B. napus* plant of Ms8 with the Bar protein; WT: wild type ZS6. The figure represented part of the results

**Fig. 3:**
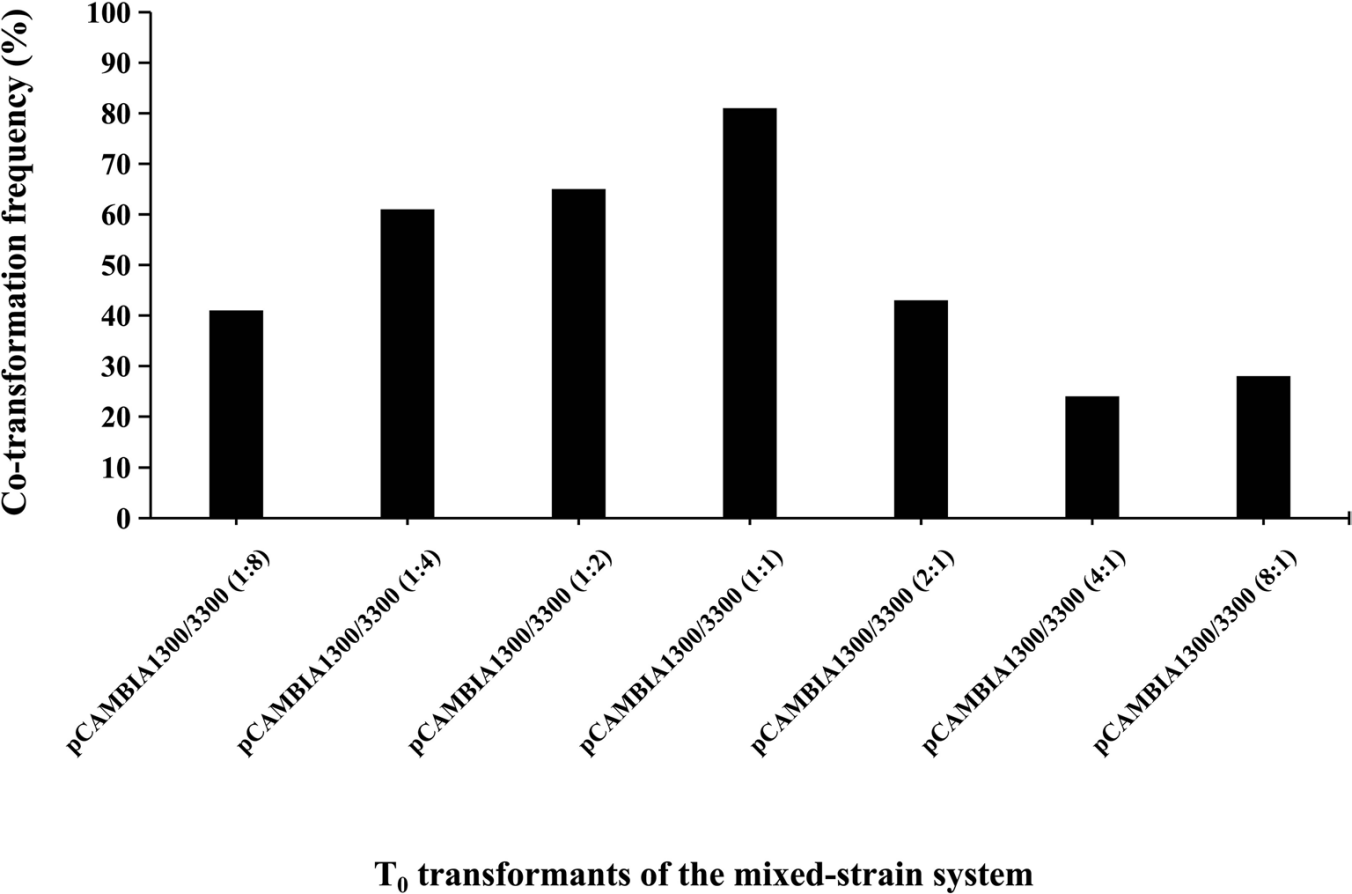
Co-transformation frequencies in T_0_ transformants of the mixed-strain system with concentration proportions of pCAMBIA1300/3300 from 1:8 to 8:1

**Fig. 4:**
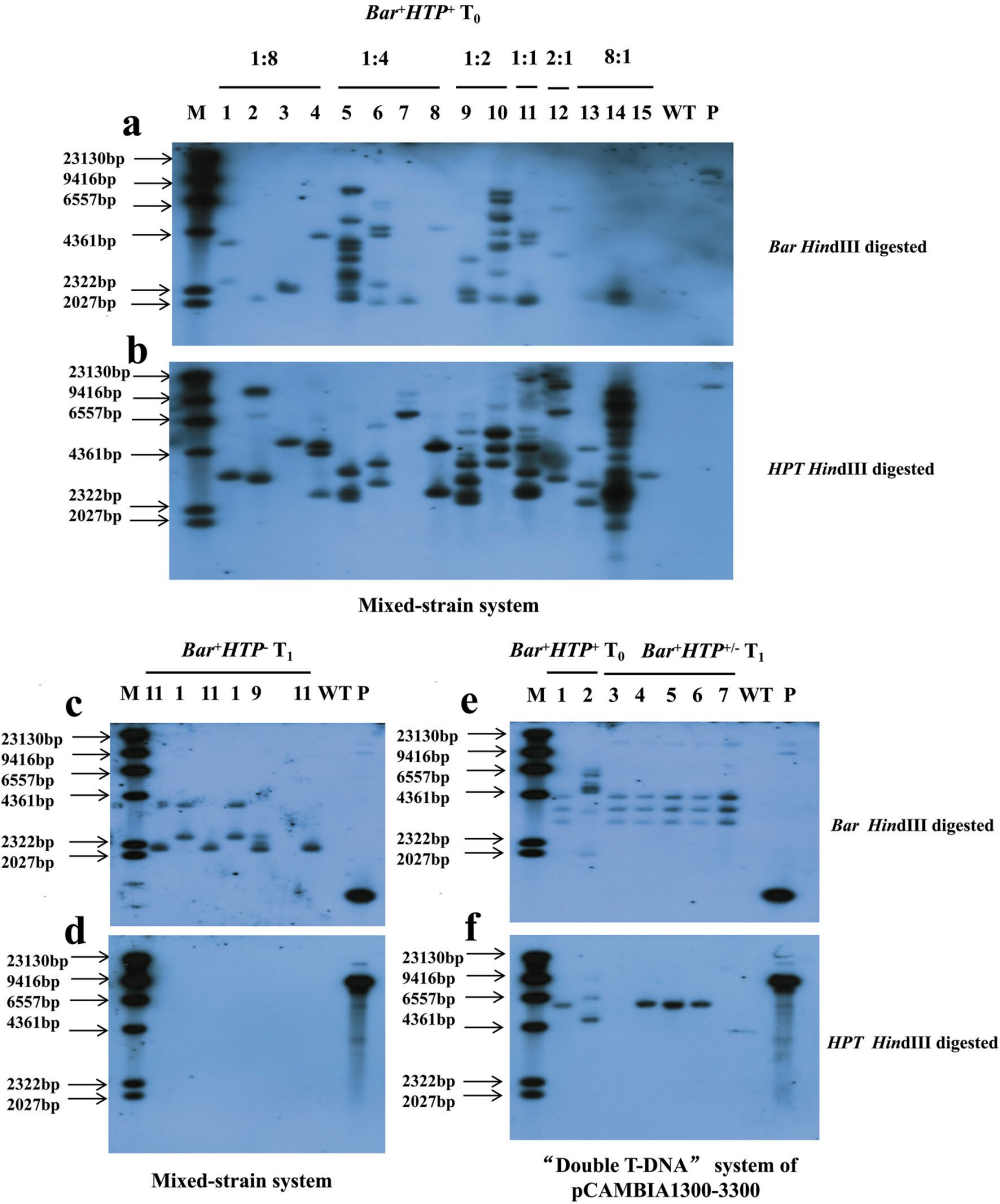
Southern blot detection in plants. *Bar* (**a**) and *HPT* (**b**) gene copy numbers were detected in T_0_ transgenic *B. napus* plants (lanes 1-15) from pCAMBIA1300/3300 of a variety of ratios (X:X) with the *Bar*^+^*HPT*^+^ genotype from the mixed-strain system. *Bar* (**c**) and *HPT* (**d**) gene were detected for identification of marker-free plants in the T_1_ generation from the mixed-strain system, 1, 9 and 11 are T_1_ progeny with the *Bar*^+^*HPT*^−^ genotype from T_0_ plants as indicated in lanes 1, 9 and 11 of **a, b**. *Bar* (**e**) and *HPT* (**f**) gene copy numbers were detected in T_0_ plants with the *Bar*^+^*HPT*^+^ genotype (lanes 1-2). *Bar* (**e**) and *HPT* (**f**) gene were detected in T_1_ individual plants including *Bar*^+^*HPT*^+^ plants and marker-free *Bar*^+^*HPT*^−^ plants from T_0_ plant as indicated in lanes 1 of **e**, **f** (lanes 3-7) from the “double T-DNA” system of pCAMBIA1300-3300. M: DNA molecular weight marker II, DIG-labelled; WT: wild type ZS6; P: expression vector pCAMBIA3300 for (**a, c, e**) and pCAMBIA1300 for (**b, d, f**)

Co-transformation frequencies of the three systems was examined in putative T_0_ transformants based on genomic PCR test strip detection. For the mixed-strain system, we tested the presence of *HPT* or/and *Bar* genes by genomic PCR (Fig. 2a, b), and performed test strip detection of the Bar protein (Fig. 2c). The detection result of individual plants from pCAMBIA1300/3300 (X:X) was shown in Table 2. Among 379 T_0_ putative root regeneration plants obtained from all transformed strains, 370 plants were confirmed to be *HPT*-positive. Among the 370 positive-tested plants, 173 plants were confirmed to have the *Bar* gene, so the average *Bar* and *HPT* gene co-transformation rate was 47% (Table 2). Our results also showed that co-transformation frequencies varied with the ratio of pCAMBIA1300/3300. With the increase of pCAMBIA1300/3300 ratio, the co-transformation frequency increased first and then decreased. It was notable that the *Bar* and *HPT* gene co-transformation frequency was 81% when the ratio of pCAMBIA1300/3300 was 1:1; this was much higher than that obtained with the other six ratios (Table 2, Fig. 3). Co-integrated T_0_ transgenic plants were identified by Southern blot to detect the integration status and copy numbers of *Bar* and *HPT* genes (Fig. 4). Blot results revealed that the *Bar* gene was a single copy in seven out of 15 (47%) T_0_*B. napus* plants (Fig. 4a lanes 2, 3, 4, 7, 8, 13 and 14). The *HPT* gene was integrated as a single copy in 3 out of 15 transgenic plants (Fig. 4b lanes 1, 3 and 15), and as three copies in most plants (Fig. 4b lanes 2, 4, 6, 7, 8 and 13). Therefore, it indicated that the *Bar* gene and the *HPT* gene were inserted into the genomes of the same transgenic plants in different copy numbers.

For the “double T-DNA” vector system of pDB1300-3300, the putative T_0_ transformants were analyzed by PCR for the insert (Fig. 2a, b lanes 20 to 22) and test strip detection for Bar protein expression (Fig. 2c lanes 11 to 17). All the 26 putative T_0_ root regeneration plants were confirmed to be *HPT*-positive by PCR, and among the 26 plants, 17 were confirmed to have the *Bar* gene. Therefore, the co-transformation frequency was 65% for the pDB1300-3300 vector (Table 2). To confirm these results, two T_0_*Bar*^+^*HPT*^+^ lines were then identified by Southern blot for detecting the integration status and copy numbers of *Bar* and *HPT* genes. Blot results revealed that the *Bar* gene had three and six copies in the two T_0_*B. napus* plants (Fig. 4e lanes 1 and 2). However, the *HPT* gene was integrated with a single copy and three copies, respectively (Fig. 4f lanes 1 and 2). The *Bar* gene and the *HPT* gene were inserted into the genomes of the same transgenic plants in different copy numbers.

**Fig. 5:**
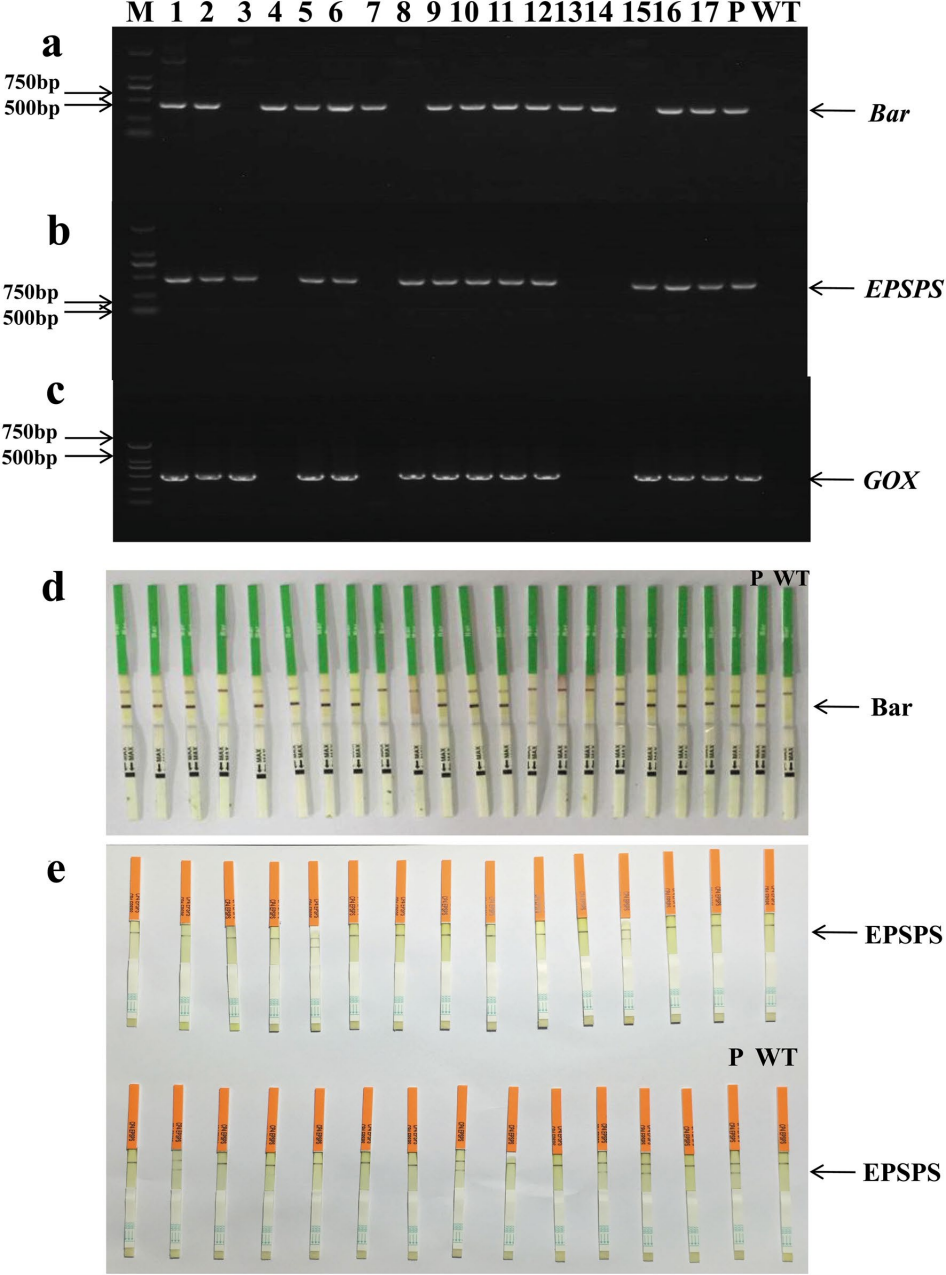
Detection of T_0_ transgenic plants from the “double T-DNA” system of pBID RT Enhanced. Putative T_0_ transformants were detected by PCR for *Bar* (**a**), *EPSPS* (**b**), and *GOX* (**c**) genes, M: DL 2000 DNA marker; 1-17: individual T_0_ plants; P: expression vector pBID RT Enhanced; WT: wild type ZS6. Putative T_0_ transgenic *B. napus* plants were detected with test strips for Bar (**d**) and EPSPS (**e**) protein, P: positive transgenic *B. napus* plant of MS8 with the Bar protein for **d** and GT73 with the EPSPS protein for **e**; WT: wild type ZS6

**Fig. 6:**
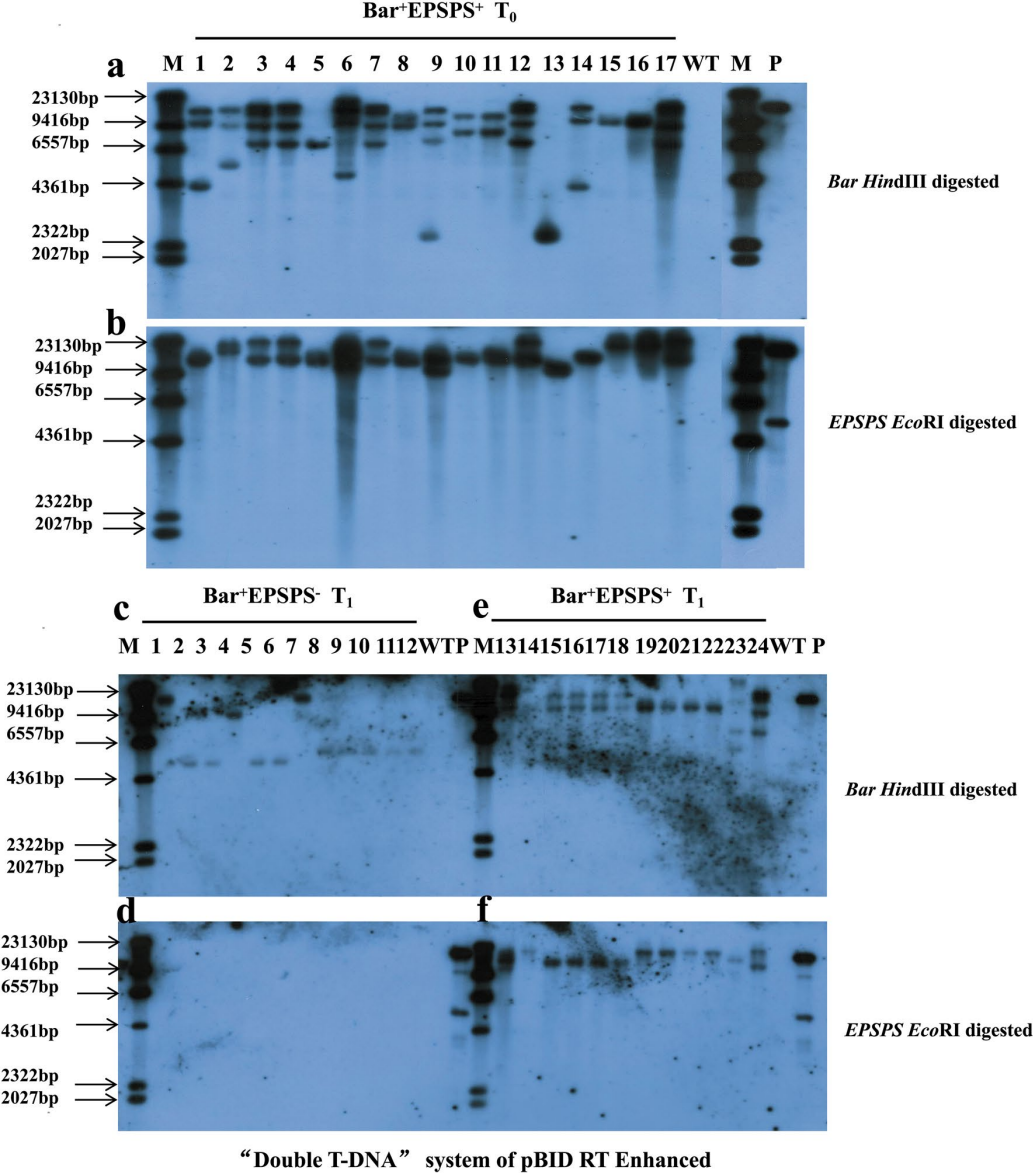
Southern blot detection in plants from the “double T-DNA” system of pBID RT Enhanced. *Bar* (**a**) and *EPSPS* (**b**) gene copy numbers were detected in T_0_ transgenic *B. napus* plants, 1-17: T_0_ individual plants with the *Bar*^+^*EPSPS*^+^ genotype. *Bar* (**c, e**) and *EPSPS* (**d, f**) gene detection for identification of marker-free and herbicide resistant plants in the T_1_ generation, 1-12: T_1_ individual plants with the *Bar*^+^*EPSPS*^−^ genotype, 13-24: T_1_ individual plants with the *Bar*^+^*EPSPS*^+^ genotype. M: DNA molecular weight marker II, DIG-labelled; WT: wild type ZS6; P: expression vector pBID RT Enhanced

For the “Double T-DNA” vector system of pBID RT Enhanced, a total of 76 putative root regeneration plants were obtained and the gene integration condition was detected by PCR for the presence of *Bar*, *EPSPS* and *GOX* genes and by test strips for the expression of Bar and EPSPS proteins (Fig. 5, Table 3). The results revealed that among 76 T_0_ putative root regeneration plants, 57 plants were confirmed to be *EPSPS*-positive. Among the 57 positive-tested plants, 18 plants were confirmed to have the *Bar* gene, so the co-transformation frequency was 32% for the pBID RT Enhanced vector (Table 3). Next, co-integrated T_0_ transgenic plants were identified by Southern blot to detect the integration status and copy numbers of *Bar* and *EPSPS* genes (Fig. 6a, b). Blot results revealed that the *Bar* gene was integrated as a single copy in four out of 17 (24%) transgenic plants (Fig. 6a lanes 5, 13, 15 and 16), and as three copies in slightly more than half plants (Fig. 6a). However, the *EPSPS* gene was a single copy in nine out of 17 T_0_*B. napus* plants (Fig. 6b). This indicated that the *Bar* gene and *EPSPS* genes were inserted in different copy number into the genomes of individual transgenic plants.

### Screening for marker-free transgenic *B. napus* plants in the T_1_ generation produced by the three systems

Marker-free efficacy was then assessed in T_1_ by frequency of marker-free lines, which was calculated as percentage of lines with marker-free plants production out of total number of identified lines, and frequency of marker-free plants in a line, which was calculated as percentage of marker-free plants out of all identified plants in a T_1_ line. For the mixed-strain system, ten T_0_ plants were randomly chosen from the 173 plants with positive *Bar* and *HPT* genes and then self-pollinated. The genotyping of every T_1_ plant at the seedling stage was performed for the presence of the *HPT* and *Bar* genes by PCR in order to identify marker-free transgenic *B. napus* plants. There were four types of segregated transgenic plants in the T_1_ generation: *Bar*^+^*HPT*^+^, *Bar*^−^*HPT*^+^, *Bar*^+^*HPT*^−^ and *Bar*^−^*HPT*^−^ (Additional file 2: Fig. S4, Table 4). Most of the plants were *Bar*^+^*HPT*^+^, and 19 marker-free plants *Bar*^+^*HPT*^−^ with resistance to phosphinothricin were identified in the six out of 10 T_1_ transgenic lines, so the frequency of marker-free lines was 60%. The frequency of marker-free plants in the 5 lines varied from 3 to 36%, with an average frequency of 11% (Table 4). To confirm these results, seven marker-free *B. napus* in T_1_ progeny with the *Bar*^+^*HPT*^−^ genotype derived from three transgenic T_0_*Bar*^+^*HPT*^+^ were selected and their genotype was verified by Southern blot with non-transformed wild-type ZS6 as a control (Fig. 4c, d). All of the seven T_1_*Bar*^+^*HPT*^−^ progeny exhibited one to three *Bar* bands without *HPT* bands and showed three different band patterns, representing these T_1_ progeny derived from three independent T_0_ transgenic lines. Two of the three T_0_ lines exhibited more than three *HPT* bands (Fig. 4b lanes 9 and 11) and these multiple bands were associated with multiple copies of the *HPT* gene in T_0_. In theory genes with multiple copies would be segregated in the next generation with a lower frequency than one-copied gene would, while these two T_0_ lines produced marker-free *B. napus* (Fig. 4c, d lanes 9 and 11) in the T_1_ generation as the T_0_ line with one *HPT* copy did (Fig. 4b lane 1 and Fig. 4c, d lane 1). In the case of hybridization with the *Bar* and *HPT* probes, one T_1_ progeny did not exhibit a signal, likely due to a false-positive PCR result.

For the “double T-DNA” vector system of pDB1300-3300, two randomly selected T_0_ plants with positive *Bar* and *HPT* genes were self-pollinated, and the T-DNA segregation in T_1_ seedlings was analyzed by testing the presence of the *HPT* and *Bar* genes by PCR. There were four types of segregated transgenic plants in the T_1_ generation: *Bar*^+^*HPT*^+^, *Bar*^−^*HPT*^+^, *Bar*^+^*HPT*^−^ and *Bar*^−^*HPT*^−^ (Additional file 2: Fig. S5, Table 4). One out of the two T_0_ transgenic lines produced five marker-free progeny *Bar*^+^*HPT*^−^ with resistance to phosphinothricin, so the frequency of marker-free lines was 50%. The frequency of marker-free plants in the line was 20% (Table 4). To confirm these results, five T_1_*B. napus* plants of one transgenic T_0_*Bar*^+^*HPT*^+^ were then identified by Southern blot (Fig. 4e, f) for confirming marker-free plants. Blot results revealed that all of the five T_1_*Bar*^+^*HPT*^−^ progeny exhibited three *Bar* bands with zero or one *HPT* bands. We observed that one of two T_0_ lines which exhibited three copies of the *HPT* gene (Fig. 4e, f lane 2) did not produce marker-free *B. napus* in the T_1_ generation. However, the other T_0_ line exhibited one copy of the *HPT* gene (Fig. 4e, f lane 1) produced marker-free *B. napus* in the T_1_ generation (Fig. 4e, f lanes 3 and 7).

For the “double T-DNA” vector system of pBID RT Enhanced, eight randomly chosen T_0_*Bar*^+^*EPSPS*^+^*GOX*^+^ plants were selfed or crossed with wild type ZS6. Seedlings of 13 T_1_ or F_1_ generations were then subjected to segregation analysis in order to identify marker-free transgenic *B. napus* plants (Additional file 2: Fig. S6, Table 5). Every T_1_ plant was tested for the presence of *Bar*, *EPSPS* and *GOX* genes by PCR. Four types of transgenic plants were detected in the T_1_ generation: *Bar*^+^*EPSPS*^+^*GOX*^+^, *Bar*^−^*EPSPS*^+^*GOX*^+^, *Bar*^+^*EPSPS*^−^*GOX*^−^ and *Bar*^−^*EPSPS*^−^*GOX*^−^ (Additional file 2: Fig. S6, Table 5). Most of the T_1_ plants were *Bar*^+^*EPSPS*^+^*GOX*^+^, and a total of 37 marker-free plants *Bar*^+^*EPSPS*^−^*GOX*^−^ were identified in the 10 out of 13 transgenic T_1_ or F_1_ transgenic lines, so the frequency of marker-free lines was 77%. The frequency of marker-free plants in the 5 selfed lines varied from 6 to 41%, with an average frequency of 19%. The frequency of marker-free plants in the 5 backcrossed lines varied from 3 to 53%, with an average frequency of 23%. The average frequency of marker-free plants in all the marker-free lines was 21% (Table 5). To confirm these results, 12 marker-free *B. napus* in the T_1_ or F_1_ generation with the *Bar*^+^*EPSPS*^−^*GOX*^−^ genotype were selected for Southern blot analysis (Fig. 6c, d). Due to linked sites of *EPSPS* and *GOX* in the same T-DNA, *EPSPS* was chosen to represent the presence of these two genes. All of the 12 T_1_*Bar*^+^*EPSPS*^−^*GOX*^−^ progeny exhibited one to two *Bar* bands without *EPSPS* band and showed four different band patterns, representing these T_1_ or F_1_ progeny derived from four independent T_0_ lines, which exhibited a single *EPSPS* copy (Fig. 6b lanes 2, 6, 8, and 15). In addition to *Bar*^+^*EPSPS*^−^*GOX*^−^ marker-free plants with resistance to phosphinothricin, *Bar*^+^*EPSPS*^+^*GOX*^+^ transgenic *B. napus* with resistance to phosphinothricin and glyphosate were obtained. Therefore, 12 of T_1_ or F_1_ progeny with the *Bar*^+^*EPSPS*^+^*GOX*^+^ genotype were chosen and verified by Southern blot with the non-transformed wild-type ZS6 used as a control (Fig. 6e, f). The 12 T_1_ or F_1_*Bar*^+^*EPSPS*^+^*GOX*^+^ progeny exhibited one to four *Bar* bands and one to two *EPSPS* bands, while showing six different band patterns, representing these T_1_ progeny derived from six independent T_0_ lines (Fig. 6e, f).

## Discussion

### Co-transformation using the three systems

In the transformation process, T-DNAs are randomly integrated into plant genomes [48]. If a transgenic plant harbors two T-DNAs in unlinked loci, genetic separation of an gene of interest from a selectable marker gene may be feasible by segregation in the successive generation [34], thereby providing a simple approach to eliminate selectable marker genes from transgenic plants. There have been successful examples of producing marker-free transgenic plants such as rice, tobacco (*Nicotiana tabacum*) and *B. napus* by co-transformation of two separate T-DNAs, one T-DNA including a gene of interest and the other a selectable marker gene [31, 49]. High efficiency of co-transformation and unlinked integration of T-DNAs with a gene of interest and a selectable marker gene in the T_1_ generation are prerequisites for the effective segregation of marker-free transgenic plants. Compared to the particle-bombardment method, which presents a very low level of effective selectable marker gene elimination due to complex and linked integrations [50, 51], *Agrobacterium*-mediated co-transformation shows simple integration patterns and is more suitable for selectable marker gene elimination. In our study, the highest frequency (81%) co-transformation was attained by using the mixed-strain method as compared with the “double T-DNA” vector system of pDB1300-3300 (65%) or pBID RT Enhanced (32%) in T_0_ plants. The co-transformation frequency of 81% is higher than most results obtained for other plant species, such as a co-transformation frequency range of 38.5 to 60.0% in wheat [19], 43.2 to 66.7% in sorghum [36] and 67% in rice [37], although there was a report that a high co-transformation efficiency of 86% in rice was achieved by placing the gene of interest into a 12-copy vector and the selectable marker gene in a single-copy co-integrated vector [52]. Another “double T-DNA” binary system developed for rice yielded 90% co-transformation, in which the first T-DNA was delimited by the *A. tumefaciens* borders and the second T-DNA was delimited by the *A. rhizogenes* borders [53].

The molar ratio of the T-DNA of the gene of interest to the T-DNA of the selectable marker gene influences the co-transformation frequency [54]. The co-transformant frequencies varied from 24 to 81% with different mixed ratios of *A. tumefaciens* strains via the mixed-strain method. Co-transformants containing both *Bar* and *HPT* genes occurred at an increasing frequency when the ratio of pCAMBIA1300 and pCAMBIA3300 was increased from 1:8 to 1:1. When the ratio of pCAMBIA1300 and pCAMBIA3300 continued to be increased from 1:1 to 8:1, the co-transformation frequency decreased. The average co-transformation frequency for the mixed-strain method was 47%, which was lower than the frequency obtained with the “double T-DNA” vector system of pDB1300-3300 (65%) but higher than that with the “double T-DNA” vector system of pBID RT Enhanced (32%). It was reported that the “double T-DNA” vector system might be more effective than the mixed-strain method in producing co-transformants [39, 40]. This view was not supported by our results, probably because co-transformation of T-DNAs from two different *Agrobacterium* strains is the equivalent of producing two independent transformation events. However, in either the “double T-DNA” vector system or the mixed-strain system, integration of each T-DNA is an independent process. Another possible reason is that the research which reported that the mixed-strain method might be less effective [39, 40] may have been unable to produce the highest co-transformation frequency due to limited mixed proportional gradients in the mixed-strain method. In their study, two mixing ratios were used, which were 1:1 and 3:1 of the T-DNA of the gene of interest to the T-DNA of the selectable marker gene, and higher co-transformation frequency was attained when the ratio was 3:1 [39, 40]. In our study, seven mixing ratios were tried—a more comprehensive approach. When compared to “double T-DNA” system, *Agrobacterium*-mediated co-transformation involving two T-DNAs on different plasmids (mixed-strain method) offers an advantage of altering to achieve the optimal ratio of T-DNA, including the selectable marker gene, to the T-DNA, including the gene of interest, to attained the highest co-transformation frequency.

In addition, the length of the T-DNA is an important element that should be considered. It has been reported that two T-DNA cassettes with different sizes in an expression vector led to different integration copy numbers in transgenic tobacco [35]. Compared with shorter T-DNA, longer T-DNA tends to have lower integration efficiency, thus affecting the final co-transformation frequency. For example, in the “double T-DNA” expression vector system of pDB1300-3300, one T-DNA region harboring shorter *Bar* genes was 1781 bp in length, and the other T-DNA containing the selectable marker gene *HPT* was 2437 bp (Fig. 1c, Additional file 2: Fig. S2). The co-transformation frequency of this vector system was 65% (Table 2). However, in the “double T-DNA” expression vector system of pBID RT Enhanced, one T-DNA region containing *Bar gene* was 1870 bp in length, but the other T-DNA region containing the selectable marker gene *CP4 EPSPS* and the *GOX V247* gene was 5775 bp, which is about three times greater (Fig. 1d, Additional file 2: Fig. S3). The co-transformation frequency of this vector system was 32% (Table 3). The incomparable size of the two T-DNAs may have resulted in fewer copies of the *CP4 EPSPS* gene than the *Bar* gene being integrated into the *B. napus* genome (Fig. 5a, b) [35]. In a “double T-DNA” vector that McCormac et al. [35] constructed, the gene of interest was placed in a shorter T-DNA and the selectable marker gene was placed in a T-DNA that was twice as long, thereby increasing integration efficiency of the unselected gene of interest. In the mixed-strain systems that Zhou et al. [39] and Komari et al. [40] reported, the T-DNA of the interested gene is 1.3 times and 1.6 times longer than that of the selected marker gene respectively, and when the molar ratio of the T-DNA of the gene of interest to the T-DNA of the selectable marker gene was 3:1, higher co-transformation frequency was attained than when the molar ratio was 1:1. Hence, it may be that if the length of two independent T-DNA regions is adjusted to an optimal ratio, stable 1:1 gene co-integration could be achieved. It is likely that it was the appropriate molar ratio based on the length ratio of the two T-DNAs that yielded a high co-transformation frequency of 81% in the mixed-strain method reported here. Further investigations could be carried out to determine how the T-DNA length affects the co-transformation frequency by setting a series of length gradients of T-DNAs.

### Marker-free situations achieved in the three systems

It has been reported that marker-free transgenic plants are more easily acquired for the progeny of T_0_ plants with a single or a few copies of selectable marker genes [19]. In our study marker-free plants tended to be obtained in some T_1_ lines where there was only one to two copies of the marker gene, i.e., the *HPT* gene in the T_0_-1 line in the mixed-strain system (Fig. 4c, d), the *HPT* gene in the T_0_-1 line in the “double T-DNA” system of pDB1300-3300 (Fig. 4e, f), and the *EPSPS* gene in the “double T-DNA” system of pBID RT Enhanced (Fig. 6c, d). Taking the pBID RT Enhanced vector as an example, the longer T-DNA cassette with *EPSPS and GOX* genes tended to integrate into the genome with fewer copies, and the shorter T-DNA cassette with the *Bar* gene tended to integrate into the genome with more copies, which were inherited with different copy numbers via independent assortment in the T_1_ generation. The efficiency of obtaining marker-free plants may indeed be closely related to the copy number of selectable marker genes.

However, marker-free plants were also obtained from T_0_ plants which had multiple copies of the marker gene. For example, marker-free transgenic plants were obtained from the two T_0_ lines despite their being five or more copies of the *HPT* marker gene in the mixed-strain system (Fig. 4 lanes 9 and 11). Theoretically we might have expected the two T_0_ lines to produce marker-free plants in the segregation generations at a very low frequency. However, it may be that the multiple copies were integrated into one or two sites in a closely linked manner, allowing marker-free plants to be more easily obtained.

### Unlinked co-insertion frequency and overall success frequency in the three systems

There were two important parameters for unlinked co-insertion frequency: the co-transformation frequency in T_0_ and the frequency of marker-free lines in T_1_. When using the mixed-strain system, co-transformation frequencies of 24 to 81% were obtained from a series of treatments (Table 2), and the frequency of marker-free lines was 60% (Table 4). Taking these collective results together, it gives a frequency of 14 to 49% (24% or 81% multiplied by 60%) of useful unlinked co-insertion events (Additional file 1: Table S2). When employing the “double T-DNA” system of pDB1300-3300, an average co-transformation frequency of 65% was obtained (Table 2), and the frequency of marker-free lines was 50% (Table 4), giving a frequency of 33% of useful unlinked co-insertion events among pDB1300-3300 transformed lines (Additional file 1: Table S2). When utilizing the “double T-DNA” system of pBID RT Enhanced, the co-transformation frequency of 32% was obtained (Table 2) and the frequency of marker-free lines was 77% (Table 4), giving a frequency of useful unlinked co-insertion events was 25% (Additional file 1: Table S2). All these results are comparable to 24% for barley [49] and 30% for rice [40]. It is difficult to compare different frequencies of independent co-insertion to other studies because of the size of the twin T-DNA binary vectors, the frequency of linked co-delivery of the double T-DNAs, and the complexity in the construction of the vectors. The *A. tumefaciens* strain types and species of the recipient plants are the major factors that affect the efficiency of these methods.

The higher positive transformant frequencies of 98% and 100% were obtained when using the mixed-strain method and the “double T-DNA” vector system of pDB1300-3300 (Table 2), and this is consistent with previous study which indicated that transformation with selectable marker gene *HPT* led to a higher positive transformant frequency (90.33%) compared with *NPTII* and *Bar* (80.23% and 65.53% respectively) [3]. The highest frequency of co-transformation of 81% was obtained when using the mixed-strain method (Table 2), probably because this method offers the advantage of altering the ratio of T-DNAs when compared to “double T-DNA” system. The highest frequency of marker-free lines of 77% were attained from the “double T-DNA” vector system of pBID RT Enhanced (Table 5) because marker-free plants are more easily produced from T_0_ plants with only one or two marker gene copies and in this system the longer T-DNA cassette including *EPSPS* and *GOX* genes tends to have lower integration efficiency. Some “double T-DNA” vectors have been constructed by inserting only “right border and left border” sequences into the polylinker of a binary vector [49]. In our study, the spacer between the two T-DNAs of the pDB1300-3300 vector was a GUS gene segment of 387 bp (Additional file 1: Fig. S2) and the distance between the two T-DNAs of the pBID RT Enhanced vector was more than 2 kb (Additional file 2: Fig. S3), and the T-DNAs were arranged in an inverted orientation. These designs may reduce the frequency of T-DNA integration into linked loci. However, two independent T-DNAs in a mixed-strain system tend to integrate into different loci in any event. The highest frequency of single copy gene of interest lines of 47% was obtained in T_0_ generation when using the mixed-strain method (Fig. 2a). The overall success in producing marker-free plants from initial transformation was summarized and taking these collective results together, it gives a overall success frequency of 7 to 22% in the mixed-strain system and 4% in the “double T-DNA” vector system of pBID RT Enhanced (Additional file 1: Table S2). Consequently it was expected that if a specific mole ratio (1:1 ratio in our study) was designed for the length ratio of the twin T-DNAs for the mixed-strain method, high unlinked co-insertion frequency and overall success frequency could be achieved.

### Three types of vector systems created two types of herbicide resistant *B. napus* materials

Herbicide resistance is one of the most important traits utilised in plant biotechnology; on the one hand used as a selectable marker during genetic engineering and on the other hand widely used as a character trait to improve agricultural efficiency by controlling weeds. Herbicide resistant crops continue to be developed by introducing genes that can confer resistance specifically to different kinds of herbicides [55]. In addition to the vector systems developed to achieve marker-free *B. napus* plants, in our study two types of herbicide resistant *B. napus* materials were simultaneously created from the segregated progeny of “double T-DNA” binary vector pBID RT Enhanced transformed T_0_ plants, including *Bar*^+^*EPSPS*^−^*GOX*^−^ plants with resistance to phosphinothricin and *Bar*^+^*EPSPS*^+^*GOX*^+^*B. napus* with resistance to both phosphinothricin and glyphosate (Fig. 6c–f). *Bar*^+^*HPT*^−^ plants with resistance to phosphinothricin were also produced from the segregated progeny of the mixed-strain system and from the “double T-DNA” binary vector of pCAMBIA1300-3300 (Fig. 4c–f). The systems in our study provided an effective and convenient approach to develop transgenic *B. napus* with various types of herbicide resistance.

## Conclusion

Removing selection markers and unnecessary vector fragments in early generations of the transgenic crop development would eliminate the biosafety concerns surrounding these sequences and facilitate the release of commercial genetically modified *B. napus* varieties [19]. In our study, three systems for co-transformation were developed, including the mixed-strain method and “double T-DNA” vector systems of pDB1300-3300 and pBID RT Enhanced, which were arranged in opposite orientations and had a different length. We were successful in producing marker-free transgenic *B. napus* plants using all three co-transformation systems and compared and evaluated these co-transformation methods. If a appropriate mole ratio was designed for the specific length ratio of the twin T-DNAs for the mixed-strain method, high unlinked co-insertion frequency and overall success frequency could be achieved. These systems provide an effective and convenient approach to developing herbicide resistant transgenic *B. napus*. The three vector systems can be used for experimental purposes, for crop improvement and for commercial needs. These systems have the potential to transform many plant species, including dicotyledonous and monocotyledonous plants in practical application.

## Supplementary information

**Additional file 1: Table** **S1.** The sequences of six units included in the artificially synthetic fragment. **Table** **S2.** The unlinked co-insertion frequency and overall success frequency in the three systems.

**Additional file 2: Fig. S1.** Structures of pCAMBIA1300 and pCAMBIA3300 that contain single independent T-DNA regions. **Fig. S2.** Construction of pDB1300-3300, which contains two independent T-DNA regions in a tandem orientation. **Fig. S3.** Construction of pBID RT Enhanced containing two independent T-DNA regions in an inverted orientation. **Fig. S4** PCR detection of *Bar* (**a**) and *HPT* (**b**) genes for screening of marker-free transgenic plants in the T_1_ generation using the mixed strains method. M: DL1000 DNA marker; 1-19: segregated T_1_ plants produced by self pollination from T_0_ co-transformants from pCAMBIA1300/3300 of a variety of ratios; P: expression vector pCAMBIA3300 for (**a**) and pCAMBIA1300 for (**b**); WT: wild type ZS6. The arrows indicated marker-free individuals **Fig. S5** PCR detection of *Bar* (**a**) and *HPT* (**b**) genes for screening of marker-free transgenic plants in the T_1_ generation from the “double T-DNA” vector pDB1300-3300. M: DL1000 DNA marker; 1-18: segregated T_1_ plants produced by self pollination from T_0_ co-transformants; P: expression vector pDB1300-3300; WT: wild type ZS6. The arrows indicated marker-free individuals **Fig. S6** PCR detection of *Bar* (*A*), *EPSPS* (*B*) and *GOX* (*C*) genes for screening of marker-free transgenic plants in the T_1_ generation. M: DL 1000 DNA marker; 1-17: individual T_1_ plants; P: expression vector pBID RT Enhanced; WT: wild type ZS6. The arrows indicated marker-free individuals.

## Data Availability

The datasets supporting the conclusions and a description of the complete protocols are included within the article.
