# Supplementary material for: Comparison of three Agrobacterium-mediated co-transformation methods for generating marker-free transgenic Brassica napus plants
Source: Plant Methods. 2020 Jun 5;16:81. doi: 10.1186/s13007-020-00628-y (PMC7275470; doi:10.1186/s13007-020-00628-y)
Supplement: Supplementary file 2 — Additional file 2: Fig. S1. Structures of pCAMBIA1300 and pCAMBIA3300 that contain single independent T-DNA regions. Fig. S2. Construction of pDB1300-3300, which contains two independent T-DNA regions in a tandem orientation. Fig. S3. Construction of pBID RT Enhanced containing two independent T-DNA regions in an inverted orientation. Fig. S4 PCR detection of Bar (a) and HPT (b) genes for screening of marker-free transgenic plants in the T1 generation using the mixed strains method. M: DL1000 DNA marker; 1-19: segregated T1 plants produced by self pollination from T0 co-transformants from pCAMBIA1300/3300 of a variety of ratios; P: expression vector pCAMBIA3300 for (a) and pCAMBIA1300 for (b); WT: wild type ZS6. The arrows indicated marker-free individuals Fig. S5 PCR detection of Bar (a) and HPT (b) genes for screening of marker-free transgenic plants in the T1 generation from the “double T-DNA” vector pDB1300-3300. M: DL1000 DNA marker; 1-18: segregated T1 plants produced by self pollination from T0 co-transformants; P: expression vector pDB1300-3300; WT: wild type ZS6. The arrows indicated marker-free individuals Fig. S6 PCR detection of Bar (A), EPSPS (B) and GOX (C) genes for screening of marker-free transgenic plants in the T1 generation. M: DL 1000 DNA marker; 1-17: individual T1 plants; P: expression vector pBID RT Enhanced; WT: wild type ZS6. The arrows indicated marker-free individuals. [file 13007_2020_628_MOESM2_ESM.docx]

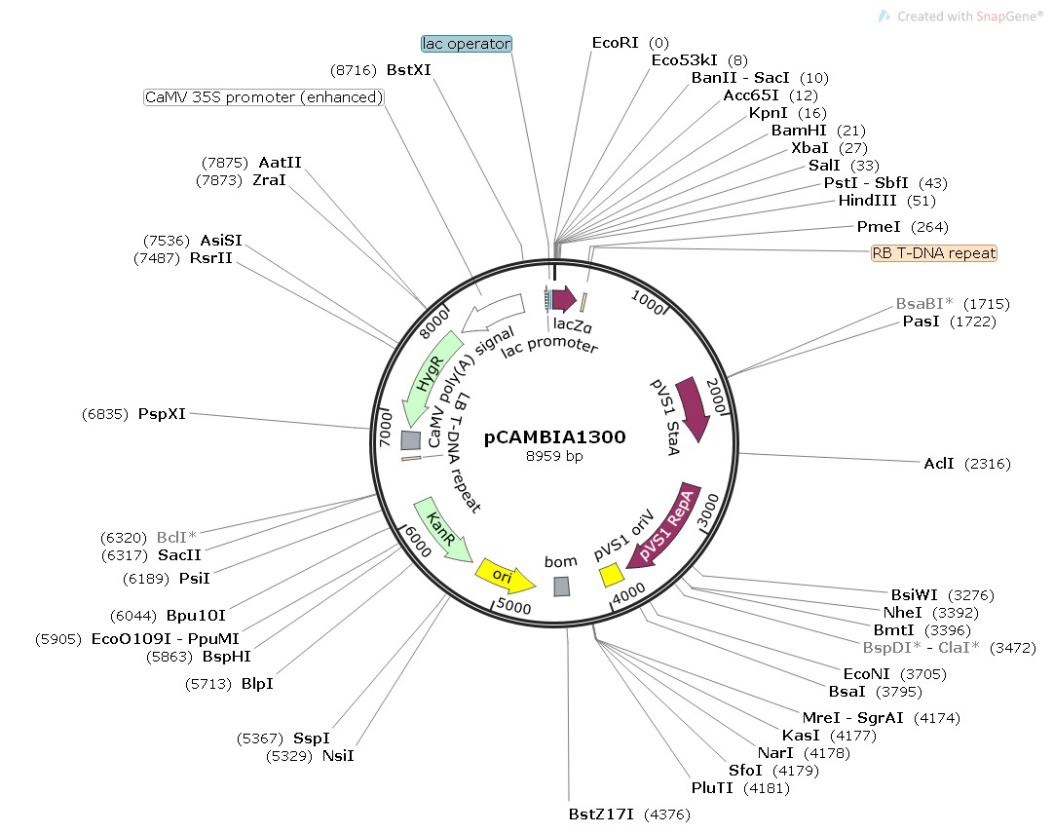


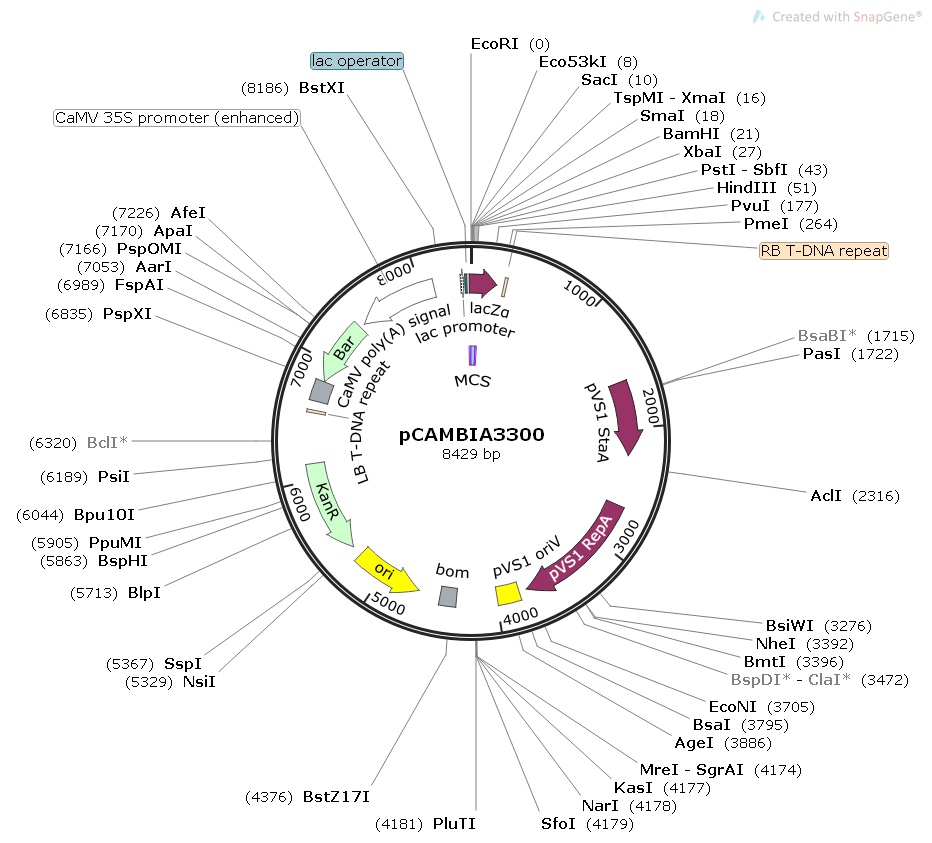


**Fig. S1** Structures of pCAMBIA1300 and pCAMBIA3300 that contain single independent T-DNA regions


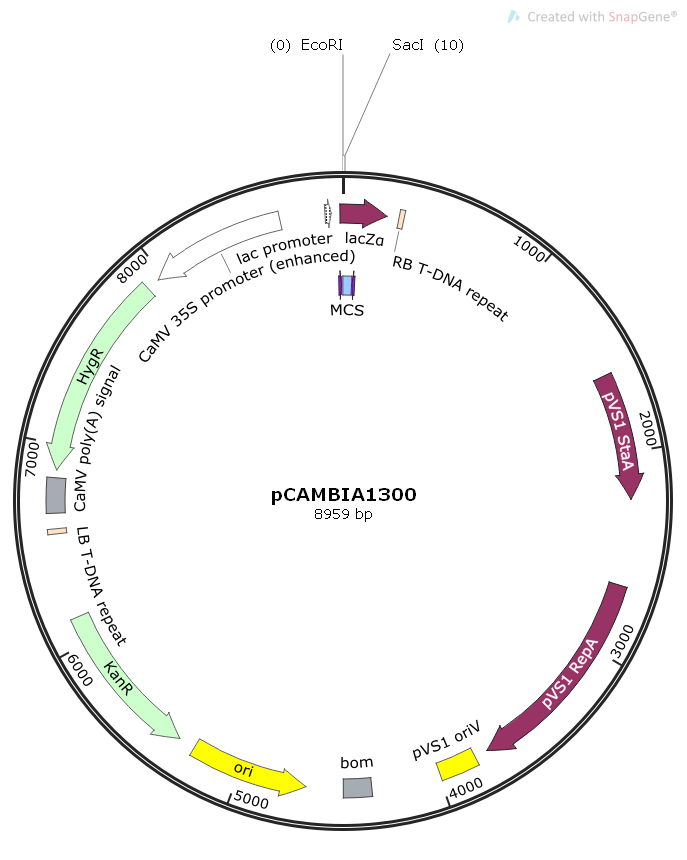

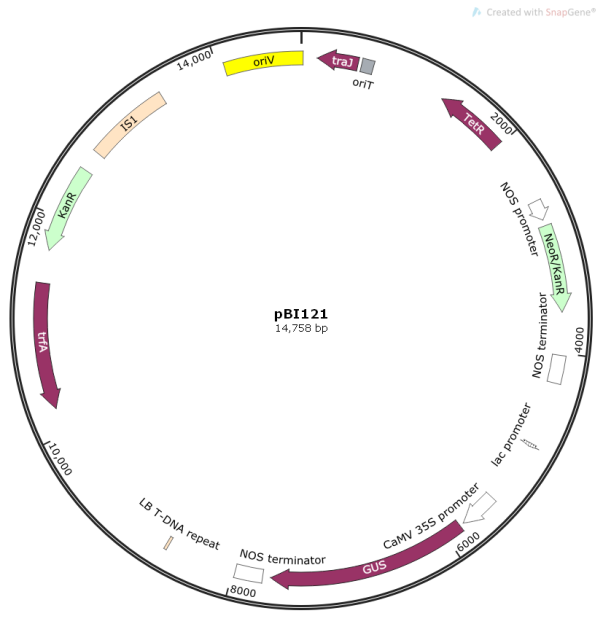


LB+GUS fragment +RB

EcoRI, SacI


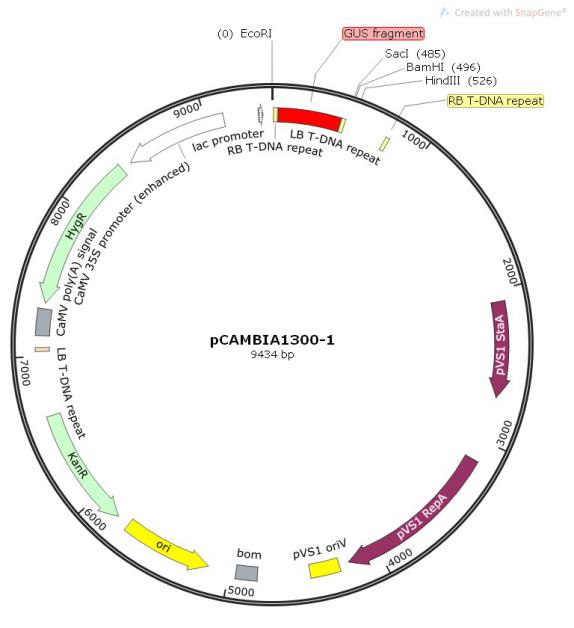

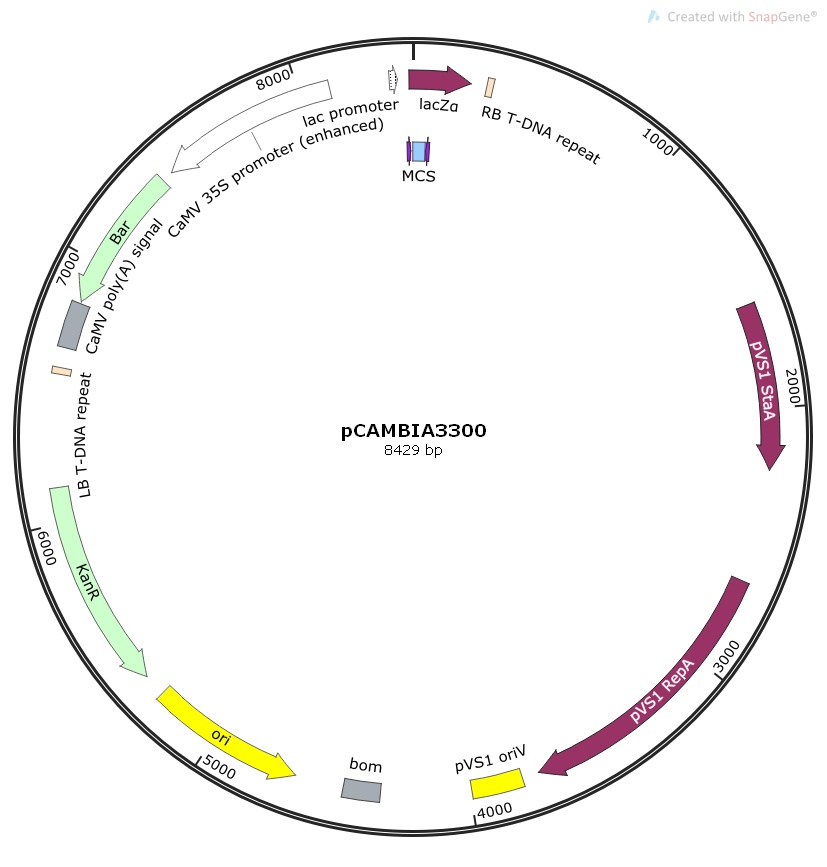


PlolyA+Bar+35S

BamHI, HindIII


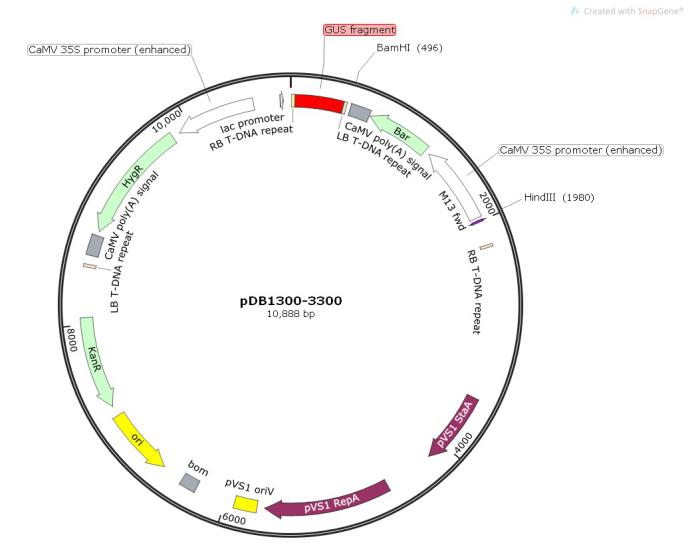


**Fig. S2** Construction of pDB1300-3300, which contains two independent T-DNA regions in a tandem orientation


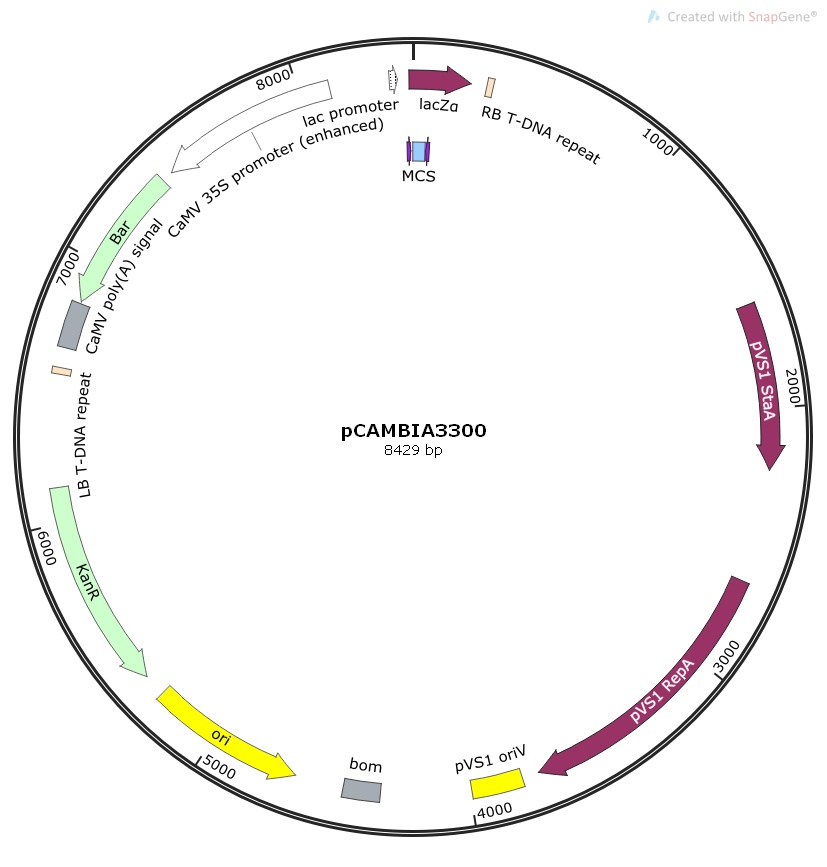


Phosphorylate the MCS and insert new MCS between pBR322 bom and pVS1 oriV sites


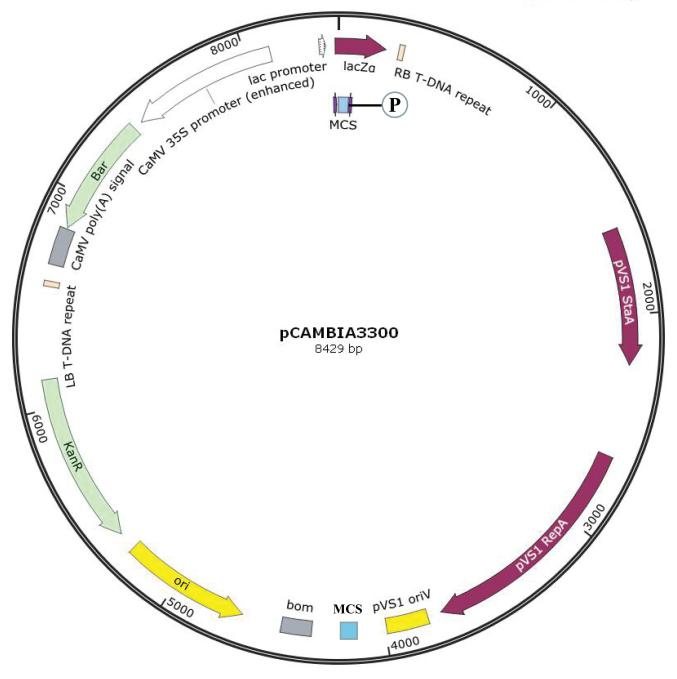


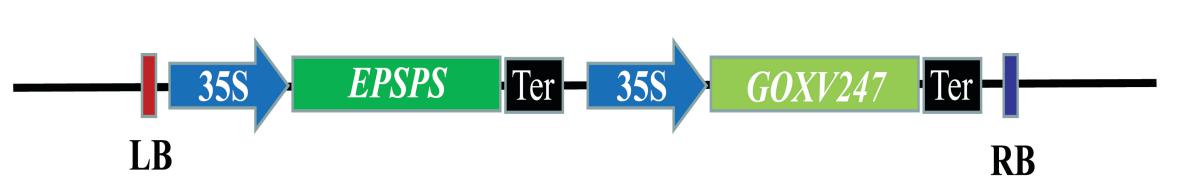


Insert into new MCS


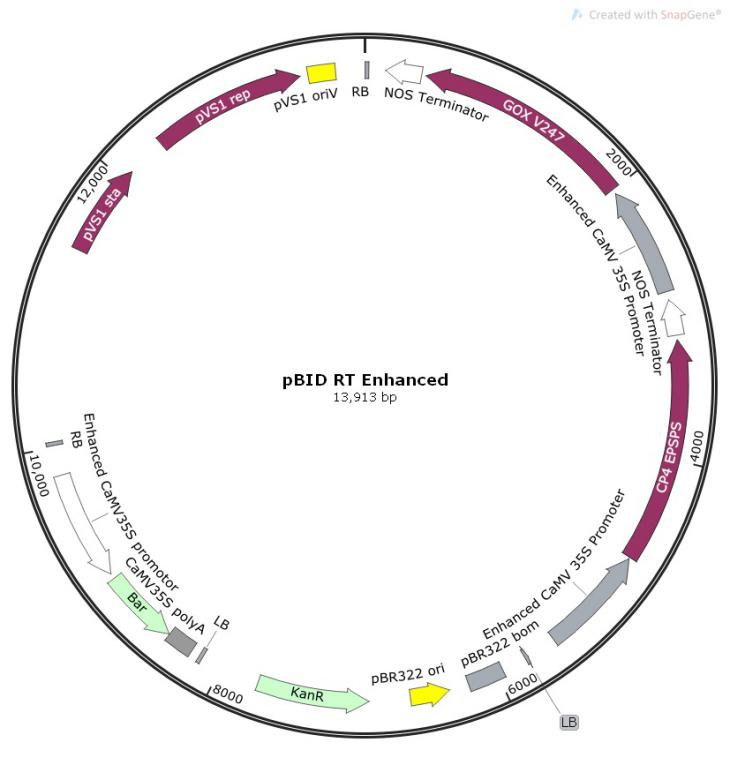


**Fig. S3** Construction of pBID RT Enhanced containing two independent T-DNA regions in an inverted orientation


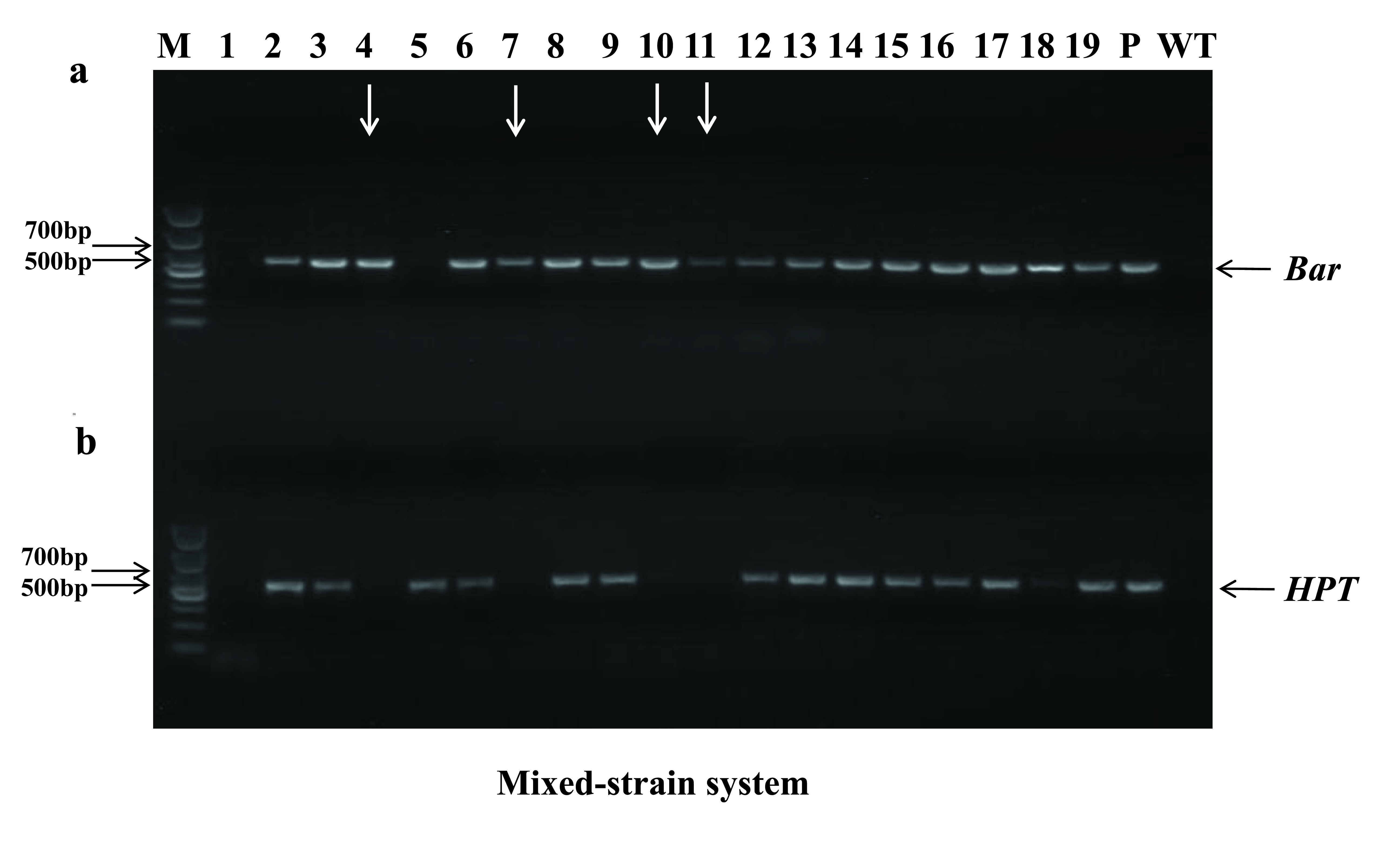


**Fig. S4** PCR detection of *Bar* (**a**) and *HPT* (**b**) genes for screening of marker-free transgenic plants in the T_1_ generation using the mixed strains method. M: DL1000 DNA marker; 1-19: segregated T_1_ plants produced by self pollination from T_0_ co-transformants from pCAMBIA1300/3300 of a variety of ratios; P: expression vector pCAMBIA3300 for (a) and pCAMBIA1300 for (b); WT: wild type ZS6. The arrows indicated marker-free individuals


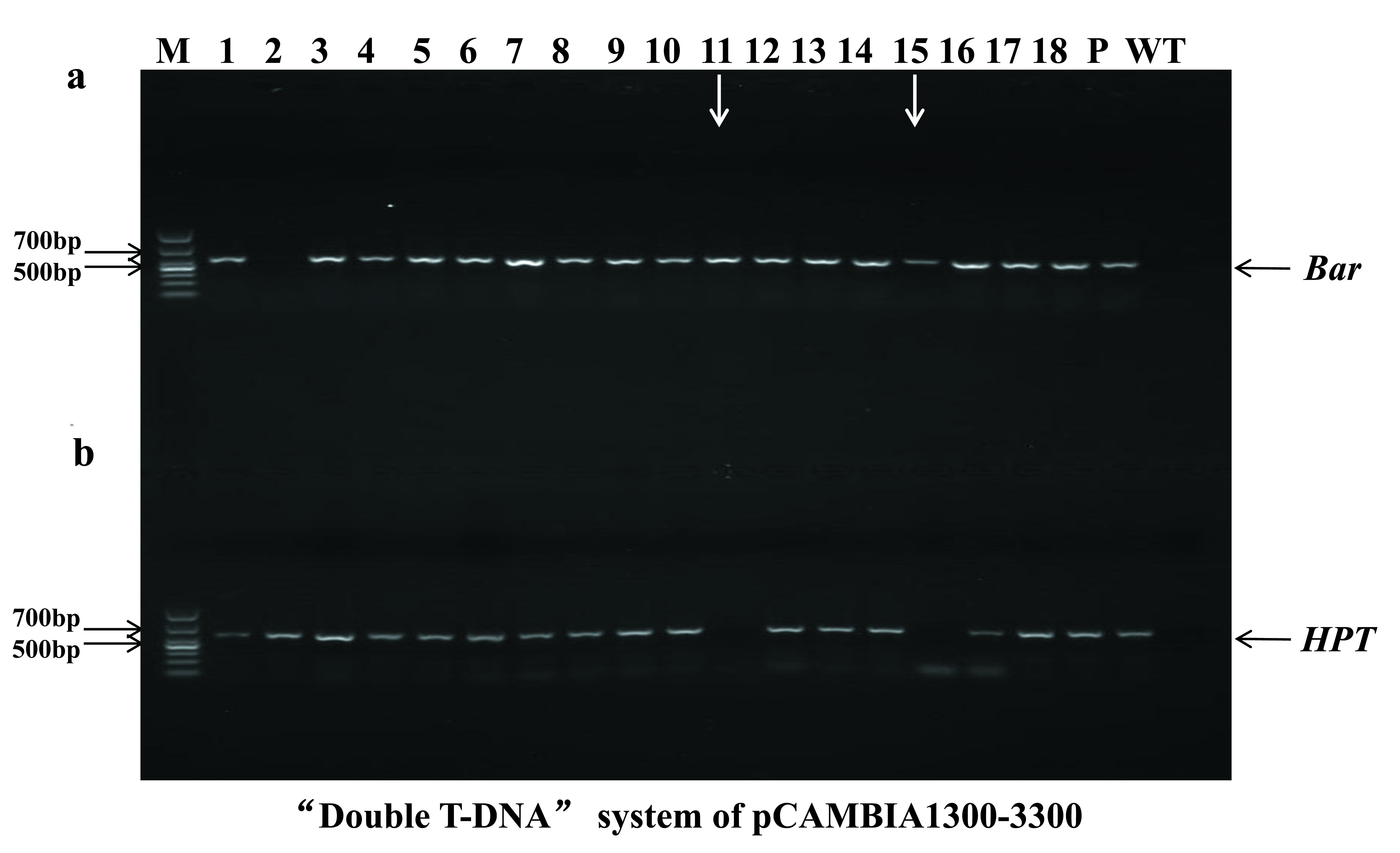


**Fig. S5** PCR detection of *Bar* (**a**) and *HPT* (**b**) genes for screening of marker-free transgenic plants in the T_1_ generation from the “double T-DNA” vector pDB1300-3300. M: DL1000 DNA marker; 1-18: segregated T_1_ plants produced by self pollination from T_0_ co-transformants; P: expression vector pDB1300-3300; WT: wild type ZS6. The arrows indicated marker-free individuals


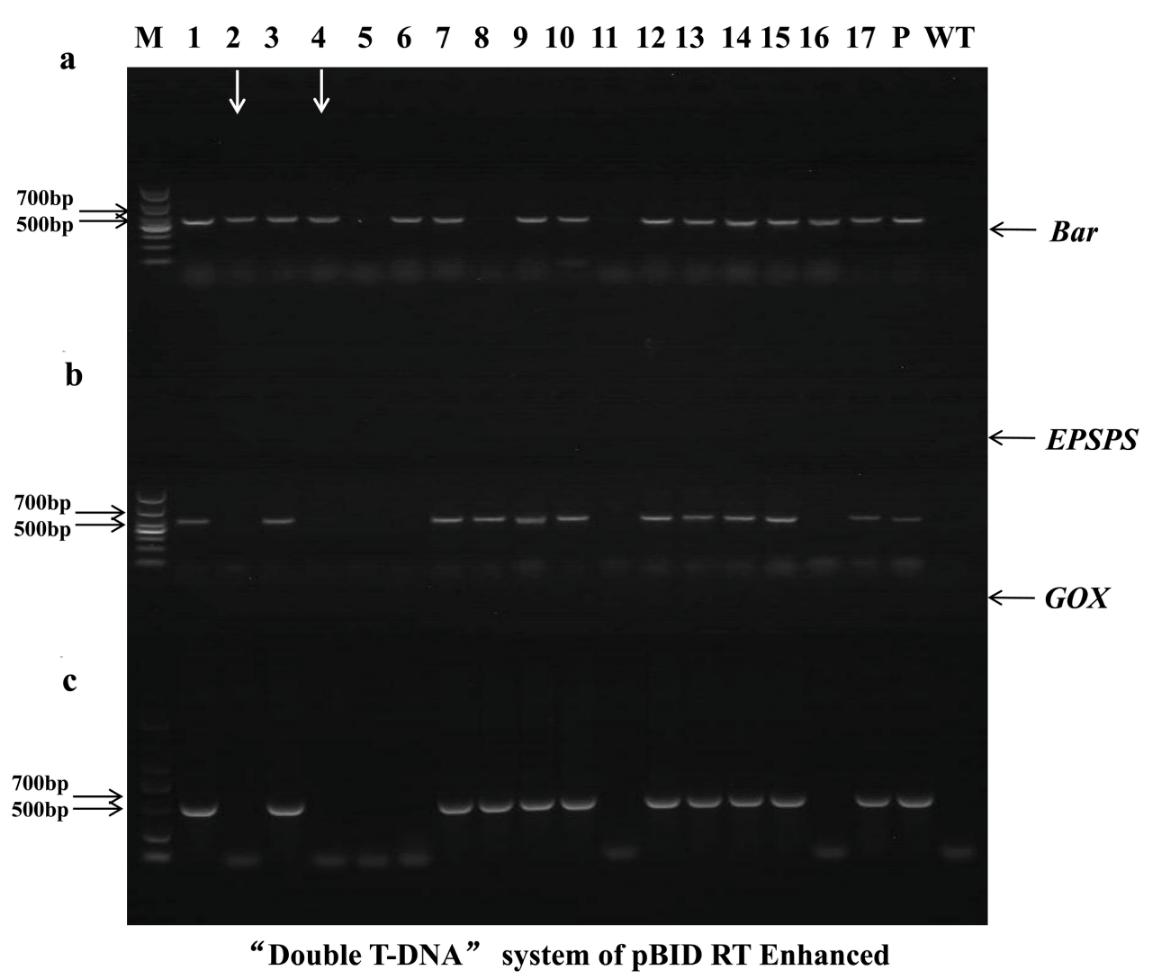


**Fig. S6** PCR detection of *Bar* (**a**), *EPSPS* (**b**) and *GOX* (**c**) genes for screening of marker-free transgenic plants in the T_1_ generation. M: DL 1000 DNA marker; 1-17: individual T_1_ plants; P: expression vector pBID RT Enhanced; WT: wild type ZS6. The arrows indicated marker-free individuals
